# Caspase-8, receptor-interacting protein kinase 1 (RIPK1), and RIPK3 regulate retinoic acid-induced cell differentiation and necroptosis

**DOI:** 10.1038/s41418-019-0434-2

**Published:** 2019-10-28

**Authors:** Masataka Someda, Shunsuke Kuroki, Hitoshi Miyachi, Makoto Tachibana, Shin Yonehara

**Affiliations:** 1 Graduate School of Biostudies, Kyoto University, Kyoto, 606-8501 Japan; 2 Graduate School of Pharmaceutical Sciences, Kyoto University, Kyoto, 606-8501 Japan; 3 Graduate School of Frontier Biosciences, Osaka University, Suita, 565-0871 Japan; 4 Institute for Frontier Life and Medical Sciences, Kyoto University, Sakyo-ku, Kyoto, 606-8507 Japan

**Keywords:** Cell biology, Development

## Abstract

Among caspase family members, Caspase-8 is unique, with associated critical activities to induce and suppress death receptor-mediated apoptosis and necroptosis, respectively. Caspase-8 inhibits necroptosis by suppressing the function of receptor-interacting protein kinase 1 (RIPK1 or RIP1) and RIPK3 to activate mixed lineage kinase domain-like (MLKL). Disruption of *Caspase-8* expression causes embryonic lethality in mice, which is rescued by depletion of either *Ripk3* or *Mlkl*, indicating that the embryonic lethality is caused by activation of necroptosis. Here, we show that knockdown of *Caspase-8* expression in embryoid bodies derived from ES cells markedly enhances retinoic acid (RA)-induced cell differentiation and necroptosis, both of which are dependent on *Ripk1* and *Ripk3*; however, the enhancement of RA-induced cell differentiation is independent of *Mlkl* and necrosome formation. RA treatment obviously enhanced the expression of RA-specific target genes having the retinoic acid response element (*RARE*) in their promoter regions to induce cell differentiation, and induced marked expression of RIPK1, RIPK3, and MLKL to stimulate necroptosis. *Caspase-8* knockdown induced RIPK1 and RIPK3 to translocate into the nucleus and to form a complex with RA receptor (RAR), and RAR interacting with RIPK1 and RIPK3 showed much stronger binding activity to *RARE* than RAR without RIPK1 or RIPK3. In *Caspase-8*-deficient as well as *Caspase-8*- and *Mlkl-*deficient mouse embryos, the expression of RA-specific target genes was obviously enhanced. Thus, Caspase-8, RIPK1, and RIPK3 regulate RA-induced cell differentiation and necroptosis both in vitro and in vivo.

## Introduction

Caspases, members of the cysteine protease family, play an essential role in the induction of apoptosis [1–4]. Caspase-8 was originally identified as an initiator caspase and mainly functions in the death receptor pathway of apoptosis. Upon ligation of a death receptor such as Fas [5, 6], Caspase-8 is recruited to a complex together with other factors including Fas, Fas-associated death domain (FADD) [7]. Within the complex, proximity-induced auto-cleavage through homo-oligomerization/dimerization catalytically activates Caspase-8. The activated Caspase-8 transmits the death signal mainly to executor caspases including Caspase-3, which then cleave various cellular proteins to complete the apoptosis-inducing process [8]. Caspase-8 is unique, with associated critical activities not only to induce apoptosis but also to suppress death receptor-mediated necroptosis [9, 10]. Caspase-8 inhibits necroptosis by suppressing the function of receptor-interacting protein kinase 1 (RIPK1) [11–13] to activate mixed lineage kinase domain-like (MLKL), an executer molecule of necroptosis [14]. Disruption of *Caspase-8* (*Casp8*) expression causes embryonic lethality in mice around embryonic day 11.5 (E11.5) [9, 15], which is rescued by depletion of either *Ripk3* or *Mlkl*, indicating that the embryonic lethality is caused by activation of necroptosis [10, 16, 17].

Retinoic acid (RA), which is a metabolic product of vitamin A, is a well-established signaling molecule that plays essential roles in various biological and physiological processes by regulating the expression of RA-specific target genes [18–20]. RA binds to a transcription complex in nucleus, which includes a pair of ligand-activated transcription factors composed of the RA receptor (RAR)-retinoic X receptor (RXR) heterodimer, to induce transcription of RA-specific target genes. There are three RAR genes (*Rara*, *Rarb*, and *Rarg*) and three RXR genes (*Rxra*, *Rxrb*, and *Rxrg*), and the heterodimeric pair binds to a DNA sequence called a retinoic acid-response element (*RARE*) [21–23]. Genes containing *RARE* in their promoters are known to be involved in diverse but interrelated biological processes, such as embryogenesis, growth, and differentiation [24]. Following the successful application of RA in the differentiation therapy of acute promyelocytic leukemia (APL), regulation of RA signaling was also related to differentiation, proliferation or apoptosis of tumor cells [25, 26].

## Materials and methods

### Mice

C57BL/6 mice were purchased from CLEA Japan. *Casp8*^−/−^ mice were generated as described previously [15]. C57BL/6 *Casp8*^+/−^ mice were bred and maintained in specific pathogen-free conditions. All experiments in this study were performed according to the guidelines for animal treatment at the institute of Laboratory Animals (Kyoto University).

### Cell culture

TT2 mouse ES cells, a kind gift from R. Kageyama (Kyoto University), were maintained on mitomycin C-treated MEFs in Dulbecco’s modified Eagle’s medium (DMEM, Gibco) supplemented with 1% fetal bovine serum (JRH Bioscience), 10% knockout serum replacement (KSR, Gibco), 2 mM l-glutamine, 0.1 mM β-mercaptoethanol, 0.1 mM nonessential amino acids (Gibco), 1 mM sodium pyruvate (Gibco), and 1000 U/ml LIF (CHEMICOM). MEFs, P19 cells, SK-N-SH cells, and HEK293T cells were cultured in Dulbecco’s modified Eagle’s medium (DMEM, Nacalai Tesque Inc.) supplemented with 10% fetal bovine serum (Sigma), 100 U/ml penicillin and 100 μg/ml streptomycin (Nacalai Tesque Inc.). HL60 cells, a kind gift from K. Inaba (Kyoto University), were cultured in suspension culture in RPMI 1640 (Nacalai Tesque Inc.). All cells were cultured at 37 °C in 5% CO_2_. All cell lines were tested for mycoplasma contamination.

### Plasmids, lentiviral expression vectors, and shRNA expression system

p*RARE3*-Luciferase and an expression vector carrying human RARα cDNA were kind gifts from A. Kakizuka (Kyoto University). Lentiviral vectors, originally provided by H. Miyoshi (RIKEN), were prepared as described previously [27]. For expression of mouse *Casp8* with sh*Casp8*-resistant silent mutations, the corresponding cDNA fragment after site-directed mutagenesis was subcloned into pCSII-PGK-MCS-IRES-Hyg. For expression of mouse *Tdg* and mouse *Ripk3* with sh*Ripk3*-resistant silent mutations, their corresponding cDNA fragments were subcloned into pCSII-PGK-3xFlag-MCS-IRES-Hyg. To generate stable shRNA-expressing cells, we utilized lentivirus vectors, pCSII-U6-MCS and pCSII-U6-MCS-puro (kind gifts from M. Matsuoka, Kumamoto University). shRNA-encoding DNA oligonucleotides were inserted into these vectors. To achieve the specific knockdown of mouse *Casp8* or *Fadd*, the tetracycline-inducible shRNA expression system (Tet-On shRNA system) with lentivirus-based vectors (pCSII-EF-TetR-IRES-puro and pCSII-U6tet-sh*Casp8* or sh*Fadd*-PGK-neo) was utilized in TT2 mouse ES and P19 cells as previously described [27].

The used target sequences of shRNA are listed in Supplementary Table S1.

### RA treatment for quantification of RA-induced genes

Cells were treated with 1 μM, 100 nM, 10 nM, or 1 nM RA (Sigma) for 24 h, and then expression levels of RA-induced genes were quantified. In the case of cells with a Tet-On shRNA system, cells were cultured with or without 1 μg/ml Dox for 4 days, and then treated with or without 1 µM RA for 24 h in the presence of Dox. To analyze expression of genes directly induced by RA, cells were treated with RA for 24 h.

### Differentiation of ES cells and P19 cells through EB formation

After Tet-On sh*Casp8* or sh*Fadd* ES cells were cultured with or without 1 μg/ml Dox for 2 days, single-cell suspensions were prepared by treatment with trypsin-EDTA (Nacalai Tesque Inc.). To form EBs, 3 × 10^3^ cells were seeded per well in low-cell-adhesion 96-well plates (Thermo SCIENTIFIC) in Glasgow’s Minimum Essential Medium (GMEM, Gibco) supplemented with 10% knockout serum replacement (KSR, Gibco), 2 mM l-glutamine, 0.1 mM β-mercaptoethanol, 0.1 mM nonessential amino acids (Gibco) and 1 mM sodium pyruvate (Gibco) (ES differentiation medium) in the presence of Dox. Two days after seeding, medium was changed to ES differentiation medium supplemented with or without 1 μM RA. After 2-day cultivation, formed EBs were transferred to collagen type I-coated chamber slides (Becton Dickinson), cultured for 4 days in ES differentiation medium supplemented with or without 1 μM RA (RA treatment was for 6 days in total), and subjected to immunohistochemical analysis. To induce significant differentiation of cells through EB formation, 6 days treatment with RA was necessary.

For RA-induced neural differentiation of Tet-On sh*Casp8* P19 cells, cells were treated with or without 1 μg/ml Dox for 4 days, and single-cell suspensions were prepared by treatment with trypsin-EDTA (Nacalai Tesque Inc.). To form EBs, 1 × 10^6^ cells were seeded per 10 cm nontreated dish (IWAKI) in DMEM (Nacalai Tesque Inc.) supplemented with 10% fetal bovine serum (Sigma), 100 U/ml penicillin, and 100 μg/ml streptomycin (Nacalai Tesque Inc.), and cultured for 2–6 days with or without 1 μM RA.

### LDH release assay

After Tet-On sh*Casp8* or sh*Casp8*/*Ripk3* ES cells were cultured with or without 1 μg/ml Dox for 2 days, single-cell suspensions were prepared by treatment with trypsin-EDTA (Nacalai Tesque Inc.). To form EBs, 1.6 × 10^5^ cells were seeded per well in nontreated 6-well plates (IWAKI) in ES differentiation medium in the presence of Dox. Two days after seeding, the medium was changed to ES differentiation medium supplemented with or without 1 μM RA and 1 μg/ml Dox. To inhibit necroptosis, cells were cultured with 30 µM Nec-1 (Enzo Life Science) thereafter. After a further 2-day cultivation with or without RA, Dox, and Nec-1, the LDH release assay was performed using a Cytotoxicity Detection Kit^PLUS^ (Roche) in accordance with manufacturer’s instructions. At least three biological experiments were carried out and data are presented as means ± SD.

### Western blot analysis and immunoprecipitation

For western blot analysis, cells were lysed in ice-cold lysis buffer (20 mM Tris-HCl, pH7.4, with 10% glycerol, 1% Triton X-100, 0.5% Nonidet P-40, 150 mM NaCl, and 1 mM EDTA) containing a protease inhibitor cocktail (Nacalai Tesque Inc.). Cell lysates were resolved by sodium dodecyl sulfate-polyacrylamide gel electrophoresis (SDS-PAGE) and analyzed by western blot analysis as described previously [27]. For immunoprecipitation, cells were lysed in RIPA buffer (50 mM Tris-HCl, pH 7.5, with 150 mM NaCl, 1 mM EDTA, 1% NP-40, and 0.5% sodium deoxycholate) containing a protease inhibitor cocktail (Nacalai Tesque Inc.), and immunoprecipitation was performed following standard protocols. Immunoprecipitates were resolved by SDS-PAGE and analyzed by western blotting.

The antibodies used for western blot analyses and immunoprecipitation in this study were anti-mouse Caspase-8 (ALX-804-447-C100, Enzo Life Science), anti-RIPK1 (610458, BD Transduction Laboratories), anti-mouse RIPK3 (ADI-905-242-100, Enzo Life Science), anti-human Caspase-8 (M032-3, MBL), anti-Flag M2 (F3165, Sigma), anti-GFP (GF200, Nacalai Tesque Inc.), anti-MLKL (MABC604, MERCK MILLIPORE), anti-RAR (M-454, Santa Cruz), anti-Histone H3 (601901, BioLegend), anti-Caspase-3 (611049, BD Transduction Laboratories), anti-Caspase-7 (551238, BD Phamingen), anti-RXRα (D-20, Santa Cruz), anti-TDG (GTX110473, Gene Tex), anti-CBP (451, Santa Cruz), and anti-Actin (MAB1501, MERCK MILLIPORE).

### Immunocytochemistry and whole-mount in situ hybridization

Cells in chamber slides were fixed with 4% paraformaldehyde (Nacalai Tesque Inc.) in PBS for 15 min and permeabilized by three successive treatments with 0.3% Triton X-100 (Nacalai Tesque Inc.) in PBS for 2 h. Cells were treated with primary antibodies for 12 h at 4 °C, washed three times with 0.05% Tween-20 in PBS, and then treated with Alexa Fluor® 488-conjugated anti-mouse IgG (Molecular Probes) for 1 h. Fixed cells were washed three times with 0.05% Tween-20 in PBS and mounted with Fluoro-KEEPER Antifade Reagent with DAPI (Nacalai Tesque Inc.). Cells were analyzed under a confocal fluorescence microscope (OLYMPUS). The antibodies for immunocytochemistry used in this study were anti-Flag M2 (F3165, Sigma) and anti-Tuj1 (MAB1637, MECK MILLIPORE). Whole-mount in situ hybridization (*n* > 5) was performed as described previously [28, 29]. We did not use randomization.

### Dual-luciferase assay

Tet-On sh*GFP* or Tet-On sh*Casp8* TT2 mouse ES cells transfected with p*RARE3*-Luciferase and pTK-*Renilla* luciferase were cultured with or without 1 μg/ml Dox for 5 days and then treated with or without 1 μM RA for 24 h. The dual-luciferase assay was performed using a dual-luciferase assay kit (Promega) in accordance with the manufacturer’s instructions. At least three biological experiments were carried out and data are presented as means ± SD.

### Quantitative reverse transcription-polymerase chain reaction (qRT-PCR)

Total RNA was extracted using Sepasol®-RNA Super G (Nacalai Tesque Inc.) according to the manufacturer’s instructions. The reverse transcription (RT) reaction was performed using a ReverTra Ace® qRT-PCR Master Mix (TOYOBO) according to the manufacturer’s instructions. RT products were analyzed using a THUNDERBIRD® qPCR Mix (TOYOBO) and the StepOne real-time PCR system (Applied Biosystems) with the primer sets listed in Supplementary Table S2 according to the manufacturer’s instructions. The expression level of each mRNA was normalized to that of mouse or human *GAPDH*. At least three biological experiments were carried out and data are presented as means ± SD.

### Flow cytometric analysis

HL60 cells expressing sh*LacZ* or sh*CASP8* were cultured with or without 1 μM RA for 3 days and then stained with FITC-conjugated anti-CD11b antibody (eBioscience) for 30 min. Flow cytometric analysis was performed with a FACS canto2 (BD Biosciences).

### ChIP analysis

ChIP analyses were performed as previously described [30]. In brief, quantitative PCR was performed using a THUNDERBIRD® qPCR Mix (TOYOBO) and the StepOne real-time PCR system (Applied Biosystems) with the primer listed in Supplementary Table S3. The antibodies for ChIP analysis used in this study were anti-Flag M2 (F3165, Sigma), anti-RAR (M-454, Santa Cruz), and anti-RIPK1 (610458, BD Transduction Laboratories). At least three biological experiments were carried out and data are presented as means ± SD.

### Rescue experiments of *Casp8*^−/−^ embryos using an RA antagonist

BMS493 (Tocris Bioscience) in DMSO (100 mM) was diluted with olive oil to a final concentration of 3 μM just before use. BMS493 (2.5 μl/g of body weight; 7.5 pmol/g of body weight) was intraperitoneally injected into pregnant *Casp8*^+/−^ female mice intercrossed with *Casp8*^+/−^ male mice at E8.5, E9.5, and E10.5 after fertilization, and E11.5 *Casp8*^−/−^ embryos were analyzed in comparison with *Casp8*^+/+^ littermates. We did not use randomization.

### In vitro binding assay of RIPKs and RARα

Expression vectors of 3xFlag-RIPK1, 3xFlag-RIPK3, and HA-RARα were transfected into HEK293T cells. Expressed recombinant proteins were purified by using PURIFICATION KIT of DDDDK-tagged protein or HA-tagged Protein (MBL). Recombinant proteins were incubated in interaction buffer, (50 mM Tris-HCl, pH 8.0 with 100 mM NaCl, 10 mM MgCl_2_, 10% glycerol, 0.3 mM DTT, and 0.1% Nonidet P-40), and co-immunoprecipitation analysis was carried out.

### Generation of CRISPER/CAS9-mediated *Mlkl* KO mice

pX330-U6-Chimeric_BB-CBh-hSpCas9 was purchased from Addgene (Addgene plasmid 42230) [31]. Oligonucleotide for targeting sequence of mouse *Mlkl* exon4 was inserted into pX330-U6-Chimeric_BB-CBh-hSpCas9 using Bbs1 site. The plasmid was microinjected into zygotes, and genomic sequence of the born mice was determined. The used target sequence of CRISPR/CAS9 system is listed in Supplementary Table S4.

### Statistical analysis

Quantitative data are presented as mean values ± SD (*n* = 3) from more than three independent repetitions. Statistical comparisons between groups were carried out with the use of one-sided Student’s *t* test. *P*-values of *p* < 0.05 (*) and *p* < 0.01 (**) were regarded to be statistically significant, and *p*-value of *p* < 0.95 was evaluated to be not significantly different (n.s.d.). We were not blinded to the group allocation during the experiment.

## Results

### Knockdown of *Casp8* expression evidently enhanced RA-induced cell differentiation

**Fig. 1:**
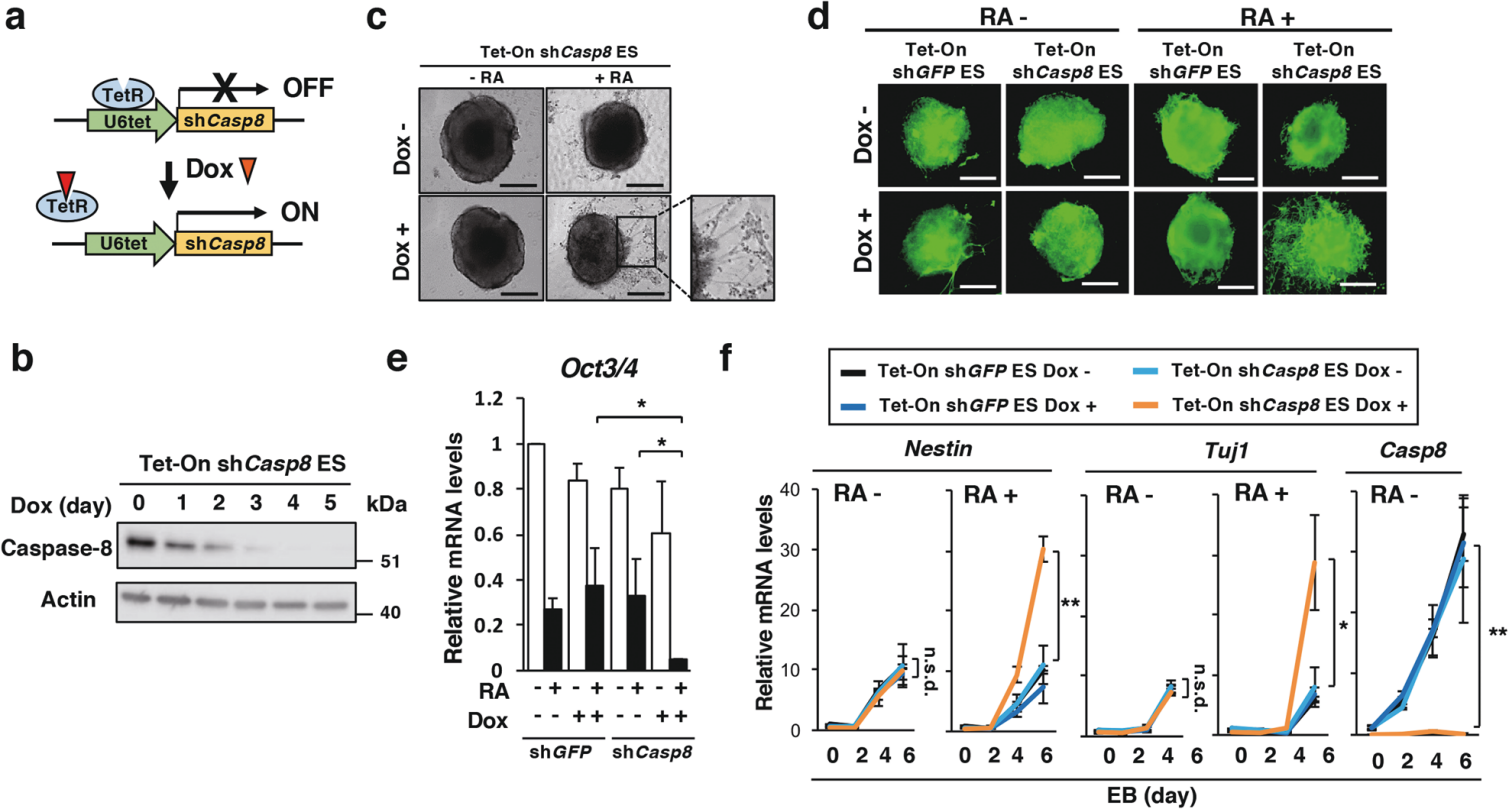
Knockdown of *Casp8* expression in ES cells enhanced RA-induced cell differentiation. **a** Dox-inducible (Tet-On) sh*Casp8*-expression system. TetR, tetracycline repressor; and U6tet, mouse *U6* promoter joining the tetracycline operator. **b** Validation of induced knockdown of *Casp8* expression in Tet-On sh*Casp8* ES cells by western blot analysis after treatment with 1 μg/ml Dox for indicated days. Molecular weight markers are indicated (kDa). Neuronal differentiation of Dox (1 μg/ml)-treated or -untreated Tet-On sh*Casp8* and Tet-On sh*GFP* ES cells was analyzed by phase-contrast microscopy (**c**) or fluorescence microscopy (**d**) after 6-day formation of EBs. EBs were treated with or without 1 μM RA for last 4 days. Scale bars, 200 μm. Cells were stained with anti-Tuj1 antibody in **d**. **e** qRT-PCR analysis of *Oct3/4* was carried out using EBs defined in **c**. **f** qRT-PCR analysis of *Nestin*, *Tuj1*, and *Casp8* was carried out using ES cells at the indicated times after formation of EBs defined in **c**. **p < 0.01, *p < 0.05 and n.s.d. (no significant difference: *p* > 0.95)

The roles of Caspase-8 on growth, viability and differentiation were investigated in mouse ES cells by utilizing a tetracycline/doxycycline (Dox)-inducible short hairpin RNA (shRNA) expression (Tet-On) system [27, 32] specific for *Casp8* (sh*Casp8*) (Fig. 1a). While *Casp8* expression was clearly downregulated by Dox treatment in ES cells with the Tet-On sh*Casp8* system (Tet-On sh*Casp8* ES cells) (Fig. 1b), significant effects on neither growth nor viability were observed in the ES cells after Dox treatment. However, 6-day RA treatment remarkably enhanced neural cell differentiation in embryoid body (EB) [33] derived from *Casp8* knockdown ES (*Casp8* KD ES) cells compared with control sh*GFP* ES cells (Fig. 1c, d). *Oct3/4*, a marker of undifferentiated cells, was strongly downregulated (Fig. 1e), and the expression levels of neural differentiation markers, *Nestin* and *Tuj1*, were upregulated in cells in RA-treated EBs derived from *Casp8* KD ES cells (Fig. 1f). Thus, knockdown of *Casp8* expression in ES cells markedly enhanced RA-induced neural differentiation.

### Knockdown of *Casp8* or *Fadd* expression markedly enhanced RA signaling

**Fig. 2:**
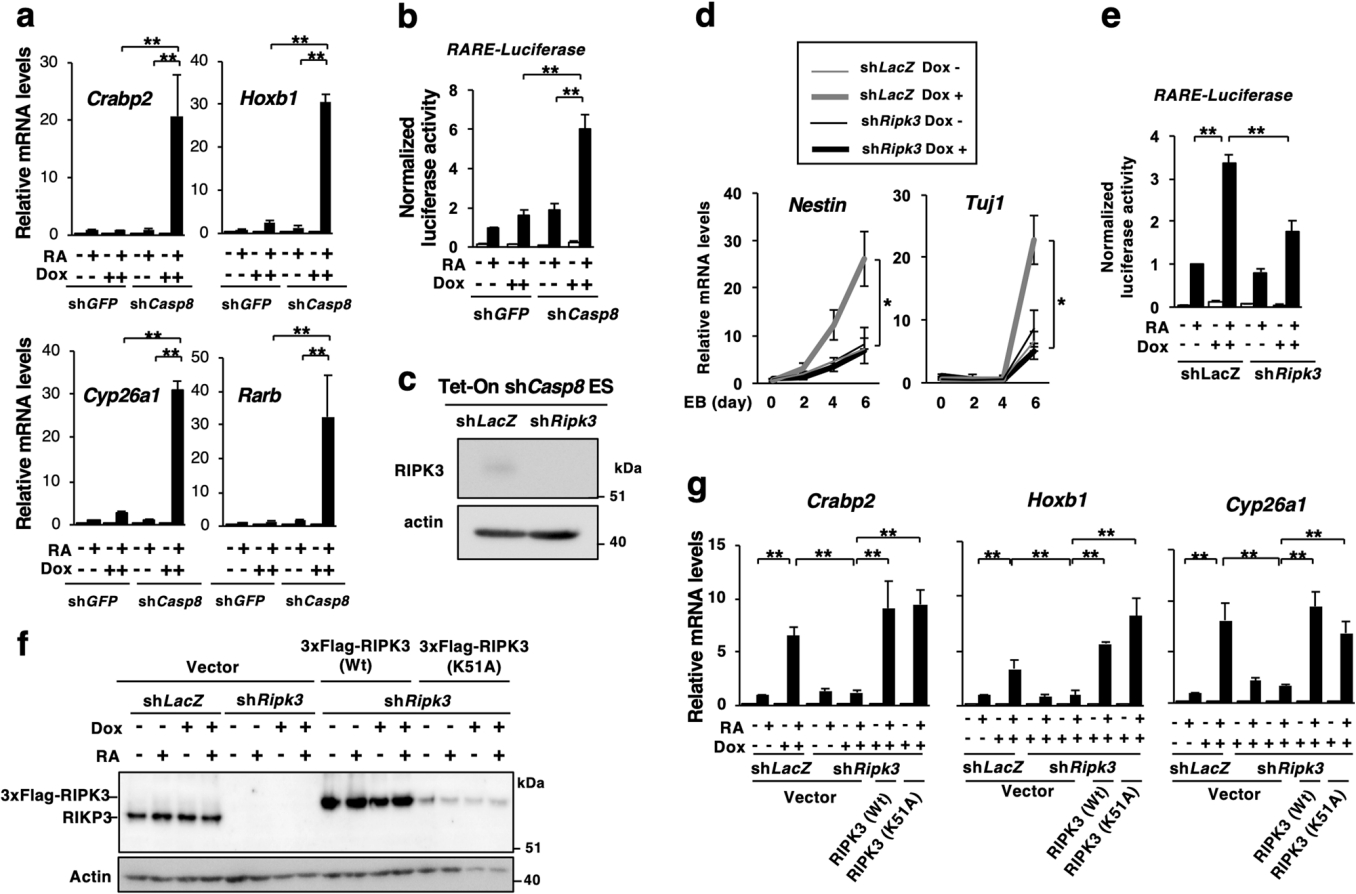
Knockdown of *Casp8* expression in ES cells markedly enhanced RA signaling dependently on RIPK3. **a** Tet-On sh*Casp8* and Tet-On sh*GFP* ES cells were cultured for 4 days with or without 1 μg/ml Dox and then treated with or without 1 μM RA for 24 h in the presence or absence of Dox. Subsequently, expression levels of RA-specific target genes, *Crabp2, Hoxb1, Cyp26a1*, and *Rarb*, were analyzed by qRT-PCR. **b** Dual-luciferase assay for *RARE* was performed using ES cells defined in **a**. **c** Western blot analysis of RIPK3 expression was carried out in Tet-On sh*Casp8* ES cells expressing sh*LacZ* or sh*Ripk3* in the absence of Dox. Actin was detected as a control. Molecular weight markers are indicated (kDa). **d** qRT-PCR analysis of *Nestin* and *Tuj1* expression was performed using EBs derived from Tet-On sh*Casp8* ES cells expressing sh*LacZ* or sh*Ripk3* after 6 days formation of EBs in the presence or absence of 1 μg/ml Dox. EBs were treated with 1 μM RA for last 4 days. **e** Dual-luciferase assay for *RARE* was carried out using Tet-On sh*Casp8* ES cells expressing sh*LacZ* or sh*Ripk3* after treatment with or without 1 μM RA for 24 h in the presence or absence of 1 μg/ml Dox. **f**, **g** Tet-On sh*Casp8* P19 cells expressing sh*LacZ* or sh*Ripk3* were infected with lentiviral vectors carrying 3xFlag-Wt *Ripk3* or K51A *Ripk3*. These cells were cultured with or without 1 μg/ml Dox for 5 days, and then treated with or without 1 μM RA for 24 h in the presence or absence of Dox. Subsequently, western blot analysis for RIPK3 (**f**) and qRT-PCR analysis for RA-inducible genes (**g**) were carried out. Vector, an empty vector. **p < 0.01 and *p < 0.05

We then analyzed the expression levels of RA-specific target genes, *Crabp2, Hoxb1, Cyp26a1*, and *Rarb*, which expressions were under the control of RARs and a *RARE* in the respective promoter regions of these genes [34–37]. qRT-PCR and dual-luciferase reporter analyses revealed that expression levels of all the RA-specific target genes and *RARE*-dependent transcription of luciferase were dramatically elevated in *Casp8* KD ES cells treated with RA (Fig. 2a, b). Because the enhanced expressions of RA-specific target genes were observed by the treatment with even 10 nM RA (Supplementary Fig. S1a), Caspase-8 suppressed evident activation of RA signaling in a physiological condition (~25 nM) [38]. In addition, the expression levels of the RA-specific target genes were similarly elevated in ES cells expressing sh*Casp8* #2, a different shRNA for mouse *Casp8* (Supplementary Fig. S1b). While one of the RA-specific target genes, which expression were enhanced by knockdown of *Casp8*, was *Rarb* (RA receptor β), the expression levels of other types of RARs than *Rarb* [39, 40], were not influenced by knockdown of *Casp8* (Supplementary Fig. S1c). Thus, Caspase-8 suppressed evident activation of RA signaling in ES cells through other mechanisms than a general increase of *RARs* expression.

The enhancement of RA signaling and RA-induced cell differentiation were induced by the inhibition of not only *Casp8* expression in mouse embryonic carcinoma cell line P19 as well as mouse ES cells, but also *Fadd* expression in ES cells (Supplementary Fig. S2). In addition, knockdown of Caspase-8 (*CASP8*) expression in human RA-sensitive cancer cell lines, SK-N-SH and HL60, clearly enhanced RA signaling and RA-induced differentiation into neural cells expressing *TUJ1* and monocytes expressing *CD11b*, respectively (Supplementary Fig. S3). Thus, Caspase-8 suppresses marked activation of RA signaling in not only mouse ES cells but also mouse embryonic carcinoma and human cancer cells.

Rescue experiments were performed using exogenous expression of knockdown-resistant wild-type (Wt) *Casp8* in *Casp8* KD ES cells, and expression of Wt *Casp8* significantly inhibited the activation of RA signaling by *Casp8* knockdown (Supplementary Fig. 4). Then, similar rescue experiments were carried out using exogenous expression of knockdown-resistant two kinds of *Casp8* mutants, CS and DE [41]. The expression of DE *Casp8*, but not CS *Casp8*, significantly inhibited the evident activation of RA signaling by *Casp8* knockdown in Tet-On sh*Casp8* P19 cells (Supplementary Fig. S4). These results showed that protease activity of procaspase-8 was indispensable but cleavage-associated activation of procaspase-8 was not necessary for inhibition of the marked activation of RA signaling.

### RIPK1 and RIPK3, but not MLKL, were involved in *Casp8* knockdown-induced enhancement of RA signaling

**Fig. 3:**
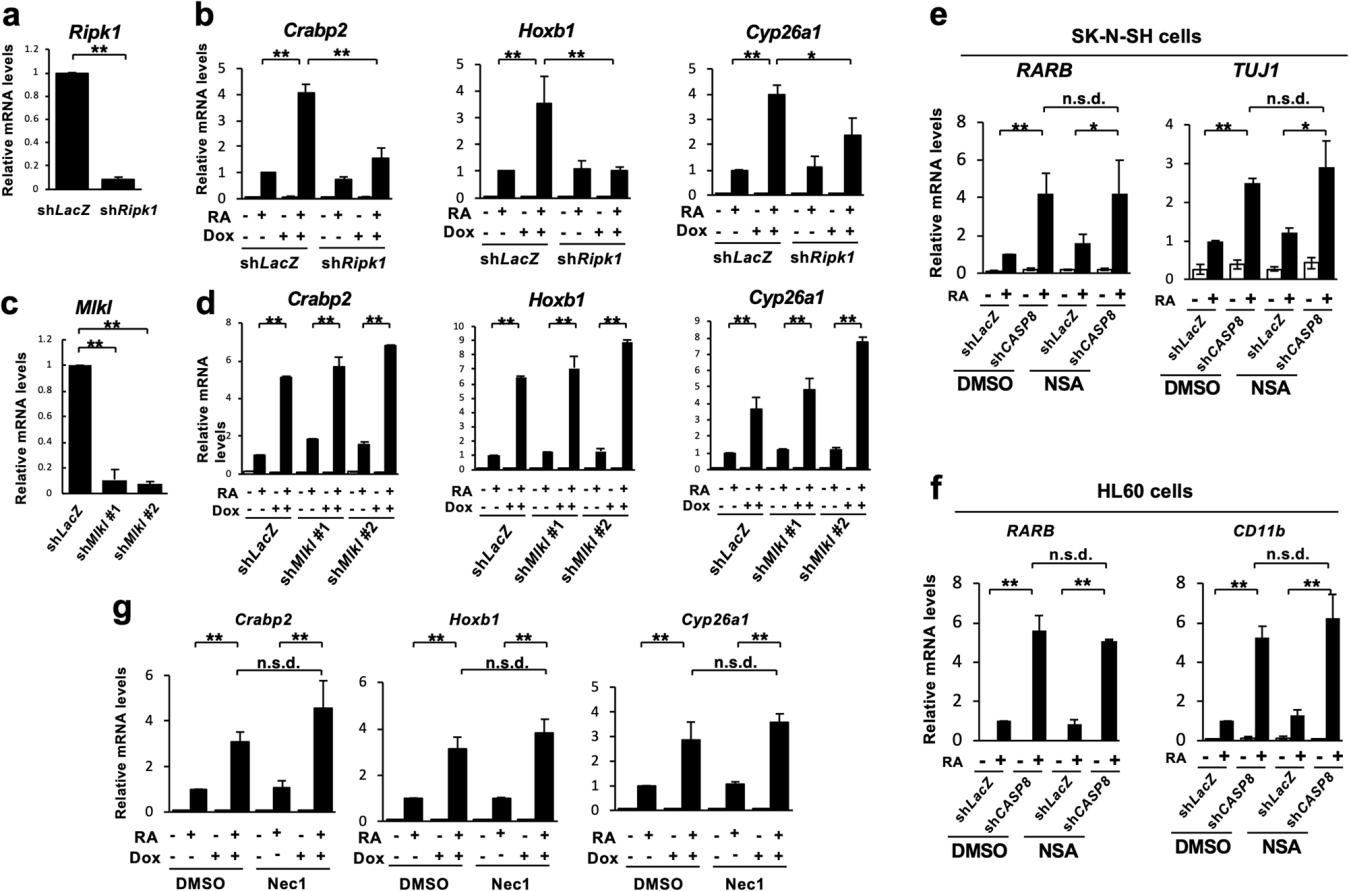
RIPK1 but not MLKL is involved in the activation of RA signaling. **a** Expression levels of Ripk1 were analyzed by qRT-PCR using Tet-On sh*Casp8* P19 cells expressing sh*LacZ* or sh*Ripk1*. **b** P19 cells defined in **a** were cultured for 4 days with or without 1 μg/ml Dox and then treated with or without 1 μM RA for 24 h in the presence or absence of Dox. Subsequently, qRT-PCR analysis of RA-induced genes, *Crabp2*, *Hoxb1*, and *Cyp26a1*, was performed. **c** Expression levels of *Mlkl* were analyzed by qRT-PCR using Tet-On sh*Casp8* P19 cells expressing sh*LacZ* or sh*Mlkl*. Two shRNAs targeting different nucleotide sequences in *Mlkl* (sh*Mlkl* #1 and sh*Mlkl* #2) were used. **d** P19 cells defined in **c** were analyzed by qRT-PCR as described in **b**. qRT-PCR analysis of *TUJ1* and *RARB*, and *CD11b* and *RARB* was carried out using SK-N-SH cells (**e**) and HL60 cells (**f**), respectively, expressing sh*LacZ* or sh*CASP8* after treatment with DMSO or 10 µM NSA for 48 h together with or without 1 μM RA for last 24 h in the presence of DMSO or 10 µM NSA. **g** qRT-PCR analysis of RA-induced genes, *Crabp2*, *Hoxb1*, and *Cyp26a1*, was performed using Dox (1 μg/ml)-treated or -untreated Tet-On sh*Casp8* P19 cells cultured with or without 1 μM RA for 24 h in the presence of DMSO or 30 μM Nec-1. **p < 0.01, *p < 0.05 and n.s.d. (no significant difference: *p* > 0.95)

Caspase-8 and FADD inhibit necroptosis mediated by RIPK1, RIPK3, and MLKL [10–14]. Downregulated expression of *Ripk3* canceled the enhancement of RA-induced differentiation in *Casp8* KD ES cells as well as the enhancement of RA-specific target genes expressions and *RARE*-dependent transcription of luciferase in *Casp8* KD P19 cells (Fig. 2c–g). In addition, *Ripk1* but not MLKL were involved in the enhancement of RA signaling in *Casp8* KD P19 cells (Fig. 3a–d). Furthermore, treatment of SK-N-SH and HL60 cells with a human MLKL-specific inhibitor, necrosulfonamide (NSA) [14], failed to inhibit the *Casp8* knockdown-induced enhancement of RA responses (Fig. 3e, f). In sh*Ripk3*-expressing *Casp8* KD P19 cells, *Casp8* knockdown-induced enhancement of RA signaling was restored by exogenous expression of knockdown-resistant Wt *Ripk3* and a kinase-negative mutant, K51A *Ripk3* [13] (Fig. 2f, g). Treatment of *Casp8* KD P19 cells with a kinase inhibitor of RIPK1, Nec-1 [42], did not inhibit the *Casp8* knockdown-induced enhancement of RA signaling (Fig. 3g). These results indicated that both RIPK1 and RIPK3 but neither MLKL nor their kinase activities played an essential role in the enhancement of RA signaling induced by the knockdown of *Casp8* expression.

RA treatment is well known to induce apoptosis. The expression levels of RA-specific target genes were not affected by double knockdown of *Casp3* and *Casp7*, which inhibited apoptosis, in either *Casp8*-expressing or *Casp8* KD P19 cells in the presence or absence of RA (Supplementary Fig. S5). These results suggested that the enhancement of RA signaling by *Casp8* knockdown was due to neither suppression nor enhancement of apoptosis.

### Knockdown of *Casp8* expression sensitized cells in EBs to RA-induced necroptosis

**Fig. 4:**
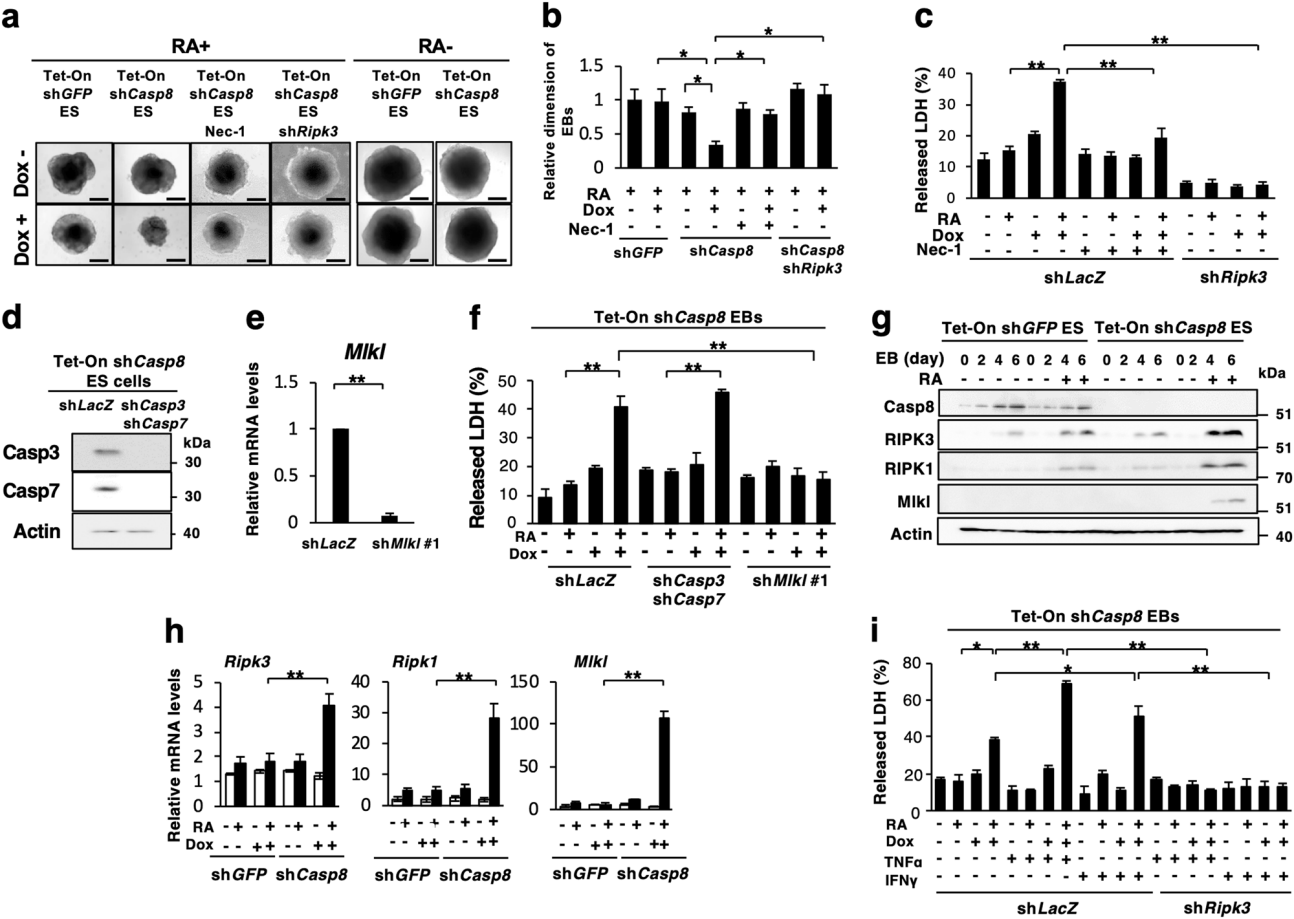
Knockdown of *Casp8* expression in ES cells enhanced RA-induced necroptosis in EBs. **a** Dox (1 μg/ml)-treated or -untreated Tet-On sh*Casp8* or Tet-On sh*GFP* ES cells expressing or not expressing sh*Ripk3* were analyzed by phase-contrast microscopy after 4 days formation of EBs. EBs were treated with or without 1 μM RA and 30 μM Nec-1 for last 2 days. Scale bars, 200 μm. **b** Relative diameters of EBs defined in **a** were measured under phase-contrast microscopy. *n* = 10. **c** Cell death was quantified by a lactate dehydrogenase (LDH) release assay after 4 days of formation of EBs derived from Tet-On sh*Casp8* ES cells expressing sh*Ripk3* or sh*LacZ*. EBs were treated with or without 1 μM RA and 30 μM Nec-1 for last 2 days. ES cells or EBs were cultured with 1 µg/ml Dox throughout the experiments. **d** Tet-On sh*Casp8* ES cells expressing sh*LacZ* or both sh*Casp3* and sh*Casp7* were subjected to western blot analysis using anti-Caspase-3 or anti-Caspase-7 antibodies. Actin was detected as a control. **e** Tet-On sh*Casp8* ES cells expressing sh*LacZ* or sh*Mlkl #1* were subjected to qRT-PCR analysis for *Mlkl*. **f** Cell death was quantified by LDH release assay after 4 days formation of EBs derived from ES cells defined in **d** and **e**. EBs were treated with or without 1 μM RA for last 2 days. *Casp8* KD ES cells or EBs were treated with 1 µg/ml Dox throughout the experiments. **g** Western blot analysis of *Casp8*, *Ripk1*, *Ripk3*, and *Mlkl* was performed using Tet-On sh*GFP* and Tet-On sh*Casp8* ES cells after 0–6 days of formation of EBs in the presence or absence of 1 μg/ml Dox. EBs were cultured with or without 1 μM RA after 3 days of formation of EBs. Actin was detected as a control. Molecular weight markers are indicated (kDa). **h** qRT-PCR analysis of *Ripk1*, *Ripk3*, and *Mlkl* was performed using ES cells defined in **g** before and after 6 days of EBs formation. **i** Cell death was quantified by LDH release assay after 4 days of formation of EBs described in **c**. EBs were treated with or without 1 μM RA for last 2 days in the presence or absence of 10 ng/ml TNFα or 10 ng/ml IFNγ. **p < 0.01 and *p < 0.05.

Two-day RA treatment of EBs derived from *Casp8* KD ES cells expressing sh*Casp8* or sh*Casp8* #2 were smaller than EBs from Tet-On sh*Casp8* ES cells treated with Dox but not with RA or those treated with RA but not with Dox (Fig. 4a, b and Supplementary Fig. S6a). We then analyzed whether cell death was induced in the EBs by LDH release assay, indicating that cell death was clearly induced in the RA-treated EBs derived from *Casp8* KD ES cells (Fig. 4c). The cell death was inhibited by treatment with Nec-1 (Fig. 4a–c). Knockdown of *Ripk3* expression, which inhibited both necroptosis and the marked enhancement of RA signaling, strongly inhibited RA-induced cell death in *Casp8* KD ES cells (Fig. 4c). RA-induced cell death in the EBs was inhibited by exogenous expression of Wt *Casp8* and DE *Casp8* but not CS *Casp8* (Supplementary Fig. S6b, c), indicating that protease activity of procaspase-8 regulated RA-induced cell death as well as RA signaling. RA-induced cell death was not inhibited in EBs derived from *Casp8* KD ES cells by the treatment with zVAD-fmk, while the cell death was slightly enhanced in EBs from *Casp8*-expressing ES cells (Supplementary Fig. S6d). Furthermore, double knockdown of *Casp3* and *Casp7* did not influence the cell death in EBs derived from *Casp8* KD ES cells; however, *Mlkl* knockdown inhibited the cell death (Fig. 4d–f). Taken together, knockdown of *Casp8* expression in EBs derived from *Casp8* KD ES cells markedly and simultaneously sensitized them to RA-induced cell differentiation and necroptosis, and effector caspases-dependent apoptosis did not influence the necroptosis in EBs.

Then the expression levels of *Ripk1*, *Ripk3*, and *Mlkl* in EBs derived from *Casp8* KD ES cells were quantified. During RA-induced differentiation, expression levels of *Ripk1*, *Ripk3*, and *Mlkl* in EBs derived from *Casp8* KD ES cells were remarkably increased in both mRNA and protein levels (Fig. 4g, h). In addition, the expression level of tumor necrosis factor α (TNFα), but not interferon α (IFNα), IFNβ or IFNγ, increased after RA treatment in EBs derived from *Casp8* KD ES cells (Supplementary Fig, S7). Importantly, TNFα and IFNγ enhanced necroptosis in RA-treated but not in RA-untreated EBs derived from *Casp8* KD ES cells (Fig. 4i). Thus, the enhancement of RA signaling by *Casp8* knockdown would sensitize *Casp8* KD cells in EBs to necroptosis through the upregulated expressions of *Ripk1*, *Ripk3, Mlkl*, and *TNFα*.

### Knockdown of *Casp8* expression induced nuclear translocation of RIPK1 and RIPK3 to form a complex with RARs

**Fig. 5:**
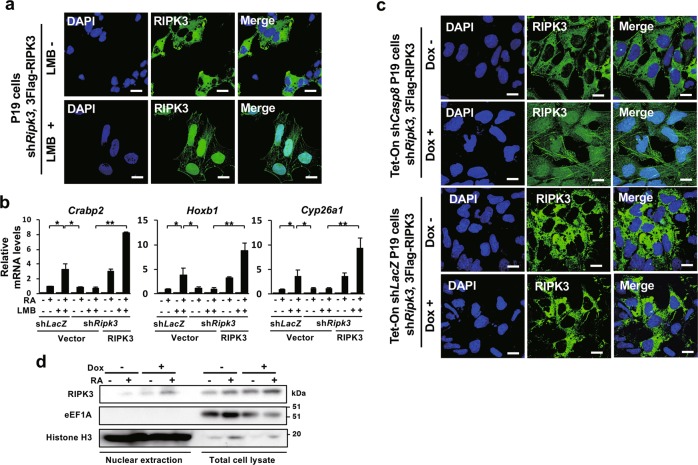
Knockdown of *Casp8* expression induced nuclear translocation of RIPK3 to enhance RA signaling. **a** P19 cells expressing sh*Ripk3* and sh*Ripk3*-resistant 3xFlag-Ripk3 were treated with or without 2 ng/ml LMB for 24 h. Subsequently, subcellular localization of 3xFlag-RIPK3 was analyzed by confocal fluorescence microscopy after staining with Alexa Fluor® 488-anti-Flag antibody and DAPI. Scale bars, 20 μm. **b** P19 cells defined in **a** and expressing sh*LacZ* were subjected to qRT-PCR analysis of RA-specific target genes, *Crabp2*, *Hoxb1*, and *Cyp26a1*, after treatment with or without 1 μM RA and 2 ng/ml LMB for 24 h. Vector, an empty vector. **c** Tet-On sh*Casp8* P19 cells and Tet-On sh*LacZ* P19 cells expressing sh*Ripk3* and sh*Ripk3*-resistant 3xFlag-Wt *Ripk3* were cultured with or without 1 μg/ml Dox for 4 days. Subsequently, subcellular localization of 3xFlag-RIPK3 was analyzed by confocal fluorescence microscopy after staining with Alexa Fluor® 488-anti-Flag antibody and DAPI. Scale bars, 20 μm. **d** Western blot analysis of endogenous RIPK3 from nuclear fractions and total cell lysates was carried out using Tet-On sh*Casp8* ES cells cultured with or without 1 μg/ml Dox for 5 days. Cells were treated with or without 1 μM RA for last 24 h. Cytoplasmic eEF1A1 and nuclear Histone H3 were simultaneously analyzed. Molecular weight markers are indicated (kDa). **p < 0.01 and *p < 0.05

Treatment of *Casp8*-expressing P19 cells with Leptomycin B (LMB), an inhibitor of nuclear export of proteins [43], converted the subcellular localization of RIPK3 from the cytoplasm to the nucleus (Fig. 5a). LMB treatment also induced promotion of RA signaling in a RIPK3-dependent manner (Fig. 5b), suggesting that RIPK3 in the nucleus might enhance RA signaling. In addition, knockdown of *Casp8* expression converted subcellular localization of RIPK3 from the cytoplasm to the cytoplasm and nucleus (Fig. 5c, d). Caspase-8 seemed to suppress nuclear translocation of RIPK3, and intranuclear RIPK3 might play an important role in the enhanced activation of RA signaling in the absence of Caspase-8.

**Fig. 6:**
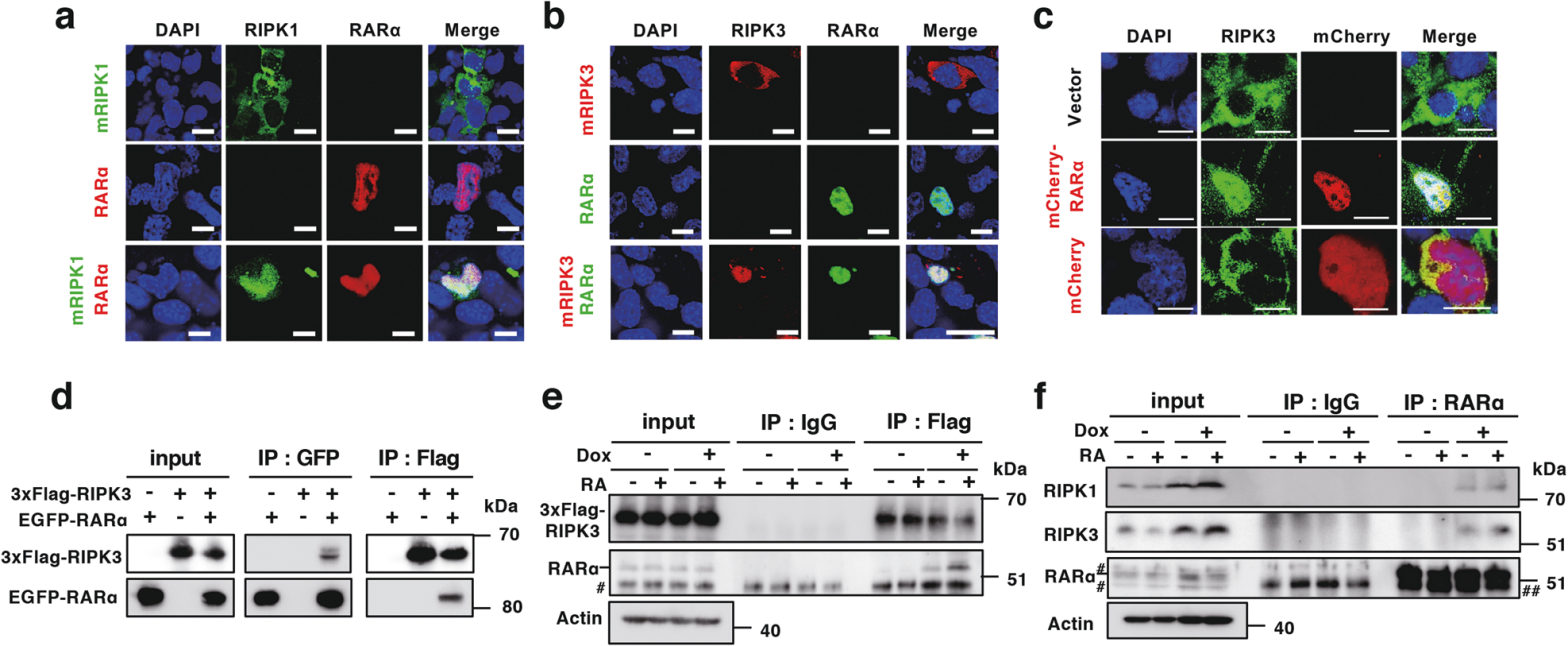
RIPK1 and RIPK3 interacted with RARα. P19 cells were transfected with expression vectors encoding EGFP-RIPK1 and/or mCherry-RARα (**a**), or mCherry-RIPK3 and/or EGFP-RARα (**b**). Cells were cultured for 48 h, and subcellular localization of these proteins was analyzed by confocal fluorescence microscopy after staining with DAPI. Scale bars, 20 μm. **c** P19 cells expressing shRipk3 and shRipk3-resistant 3xFlag-Ripk3 were transfected with an expression vector encoding mCherry-RARα or mCherry, and cultured for 48 h. Then, subcellular localization of mCherry-RARα or mCherry was analyzed by fluorescence microscopy after staining with DAPI. Scale bar, 20 µm. **d** Lysates of HEK293T cells transiently expressing 3xFlag-tagged RIPK3 and/or EGFP-RARα were subjected to immunoprecipitation (IP) with anti-Flag antibody (Flag) or anti-GFP antibody (GFP), and analyzed by western blotting with anti-Flag antibody or anti-GFP antibody. Total cell lysates (input) were also analyzed. Molecular weight markers are indicated (kDa). Dox (1 μg/ml)-treated or -untreated Tet-On sh*Casp8* ES cells expressing (**e**) or not expressing (**f**) sh*Ripk3* and sh*Ripk3*-resistant 3xFlag-Ripk3 were analyzed after 4 days formation of EBs. EBs were treated with or without 1 μM RA for last 2 days. Western blot analysis was carried out for immunoprecipitates with control IgG (IgG) or ant-Flag antibody (Flag) (**d**), and with control IgG or anti-RARα. Total cell lysates (Input) were also analyzed. Hash indicates nonspecific bands and double hashindicates IgG-derived bands

Subcellular localization of exogenously expressed RIPK1 and RIPK3 in P19 cells was also converted from the cytoplasm to the cytoplasm and nucleus by overexpression of RARα, which is a nuclear protein (Fig. 6a–c). Overexpressed RARα seemed to retain RIPK1 and RIPK3 in the nucleus. In co-immunoprecipitation experiments, exogenously expressed RIPK3 interacted with exogenously expressed RARα in HEK293T cell extracts (Fig. 6d), and immunoprecipitation of exogenous Flag-tagged RIPK3 with anti-Flag antibody co-precipitated endogenous RARα from extracts of *Casp8* KD ES cells (Fig. 6e). Importantly, endogenous RIPK1 and RIPK3 were co-immunoprecipitated with endogenous RARα from extracts of *Casp8* KD ES cells, and the interactions of RARα to both RIPK1 and RIPK3 were shown to be enhanced by RA treatment (Fig. 6f). Thus, knockdown of *Casp8* expression induced nuclear localization of RIPK1 and RIPK3, and RIPK1 and RIPK3 interacted with RARα in the nucleus.

Co-immunoprecipitation analyses using various deletion mutants of RARα and RIPK3 indicated that RARα and RIPK3 interacted through the ligand-binding domain (LBD) of RARα and the protein kinase domain (PKD) of RIPK3 (Supplementary Fig. S8a–d). In vitro binding assay also revealed that both RIPK1 and RIPK3 could directly interact with RARα (Supplementary Fig. S8e, f). Typical nuclear localization signals (NLSs) were found in RIP homotypic interaction motif (RHIM) domains of both RIPK1 and RIPK3 (Supplementary Fig. S9). RHIM domains of RIPK1 and RIPK3 were localized in nucleus and these RHIM domains seemed to co-localize with RARα in the nucleus (Supplementary Fig. S10a, b). Moreover, an AAAA mutation in RHIM domains of RIPKs restricted the nuclear localization of RIPKs (Supplementary Fig. S10a, b). In co-immunoprecipitation experiments revealed that RHIM domain of RIPK3 was important for interaction between RIPK3 and RARα (Supplementary Fig. S10c, d). In sh*Ripk3*-expressing *Casp8* KD P19 cells, *Casp8* knockdown-induced enhancement of RA signaling was completely suppressed by exogenous expression of knockdown-resistant RHIM domain AAAA mutant RIPK3 (Supplementary Fig. S10e). Taken together, RIPK1 and RIPK3 independently and directly bind to LBD of RARα through their PKDs, and RHIM domains of RIPKs containing NLS are essential for both binding of RIPK3 to RARα and *Casp8* knockdown-induced enhancement of RA signaling.

### RIPK1 and RIPK3 in the transcriptional coactivator complex has a functional role to enhance RA-dependent transcription

**Fig. 7:**
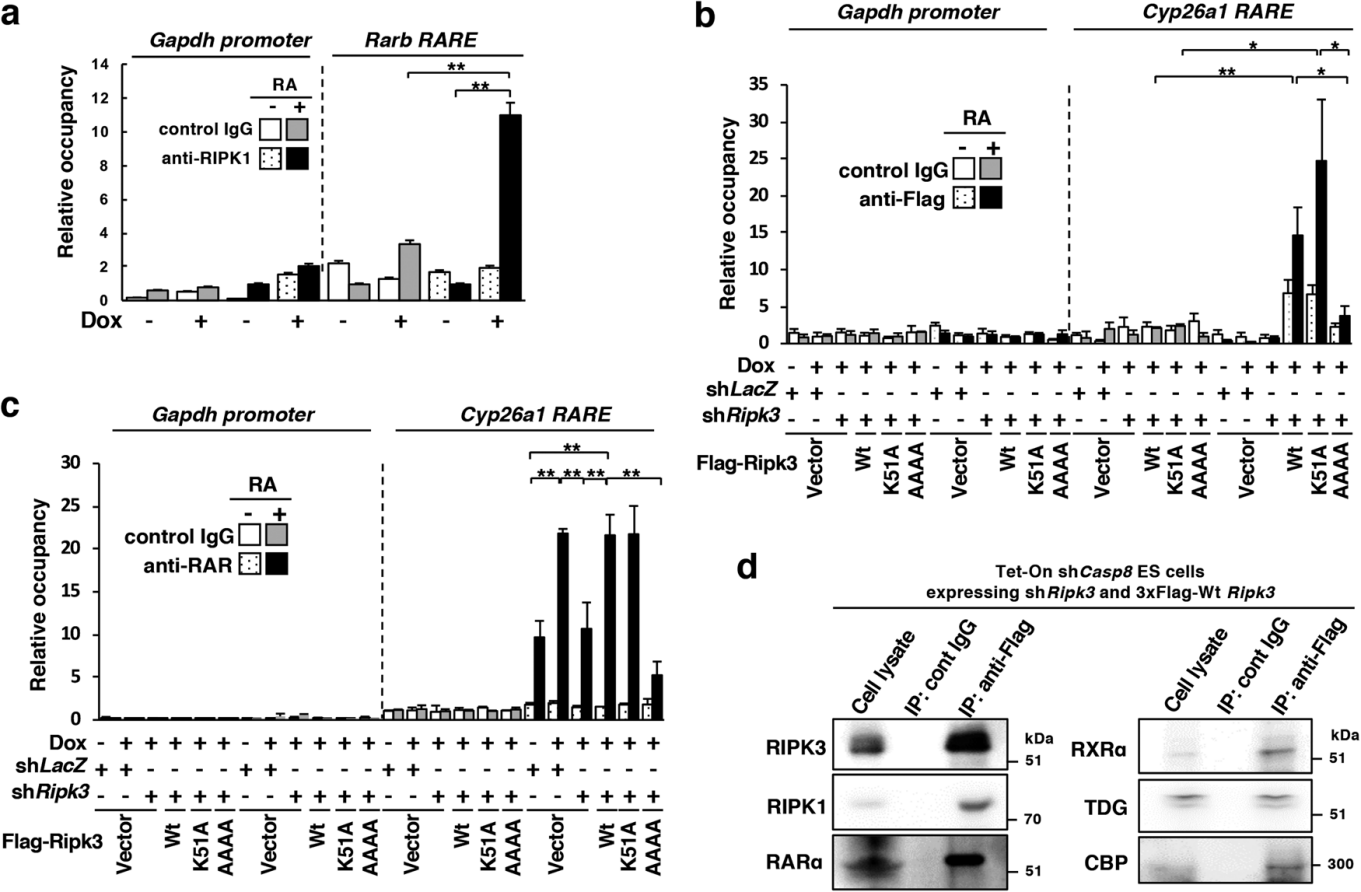
RA treatment notably enhanced binding of not only RARs but also RIPK1 and RIPK3 to *RARE*s of RA-inducible genes in the absence of *Casp8* expression. **a** Tet-On sh*Casp8* ES cells were cultured with or without 1 μg/ml Dox for 4 days and then treated with or without 1 μM RA for 24 h in the presence or absence of Dox. Subsequently, ChIP analysis for the *Rarb*-specific *RARE* or *Gapdh promoter* region using an anti-RIPK1 antibody was carried out. Tet-On sh*Casp8* P19 cells expressing sh*Ripk3* (sh*Ripk3* + ) or sh*LacZ* (sh*Ripk3*-) together with or without the expression of sh*Ripk3*-resistant 3xFlag-Wt RIPK3, kinase-negative 551 A mutant RIPK3, or RHIM AAAA mutant RIPK3 were treated with or without 1 μM RA for 24 h in the presence or absence of 1 μg/ml Dox, and subjected to ChIP analysis for the *Cyr26a1*-specific *RARE* or *Gapdh promoter* region using anti-Flag antibody or control IgG (**b**), or using anti-RAR antibody or control IgG (**c**). Vector, an empty vector. **d** Dox (1 μg/ml)-treated Tet-On sh*Casp8* ES cells expressing sh*Ripk3* and sh*Ripk3*-resistant 3xFlag-Wt RIPK3 were subjected to immunoprecipitation (IP) after 4 days formation of EBs. EBs were treated with 1 μM RA for last 2 days. Immunoprecipitates with control IgG and ant-Flag antibodies were subjected to western blot analysis using indicated antibodies. Molecular weight markers are indicated (kDa). **p < 0.01 and *p < 0.05

To clarify whether the complex of RARs with RIPK1 and RIPK3 functions in RA-dependent transcription, chromatin immunoprecipitation (ChIP) analysis was carried out in *Casp8* KD ES cells. RA treatment notably enhanced binding of endogenous RIPK1 to *RARE* of an RA-inducible gene, *Rarb*, specifically in the absence of *Casp8* expression (Fig. 7a). RA treatment also enhanced the binding of exogenously expressed Wt and a kinase-negative mutant, K51A, RIPK3, but not of RIPK3 with the RHIM AAAA mutant, to *RARE* of an RA-inducible gene, *Cyp26a1*, in the absence of endogenous *Ripk3* and *Casp8* expressions (Fig. 7b). Furthermore, knockdown of *Casp8* significantly enhanced RA-induced binding of RARs to *RARE* dependently on expression of either RIPK3 or RHIM domain of RIPK3 but independently on the kinase activity of RIPK3 (Fig. 7c). Taken together, RARs, bound to RIPK1 and RIPK3 in *Casp8* KD cells, showed much stronger binding activity to *RARE* in the presence of RA than RARs without RIPK1 and RIPK3.

Knockdown of RXRα, TDG, p300, and CBP, all of which were reported to form a transcriptional coactivator complex with RAR on *RAREs* and to enhance RA signaling [44–46], inhibited the *Casp8* knockdown-induced enhancement of RA signaling (Supplementary Fig. S11) and formed a complex with RIPK3 in the nucleus of RA-treated *Casp8* KD EBs (Fig. 7d). This transcriptional coactivator complex formation with RIPK1 and RIPK3 would play an important role in the enhancement of RA-dependent transcription.

### RA signaling was enhanced in *Caspase-8*-deficient mouse embryos regardless of *Mlkl* expression

**Fig. 8:**
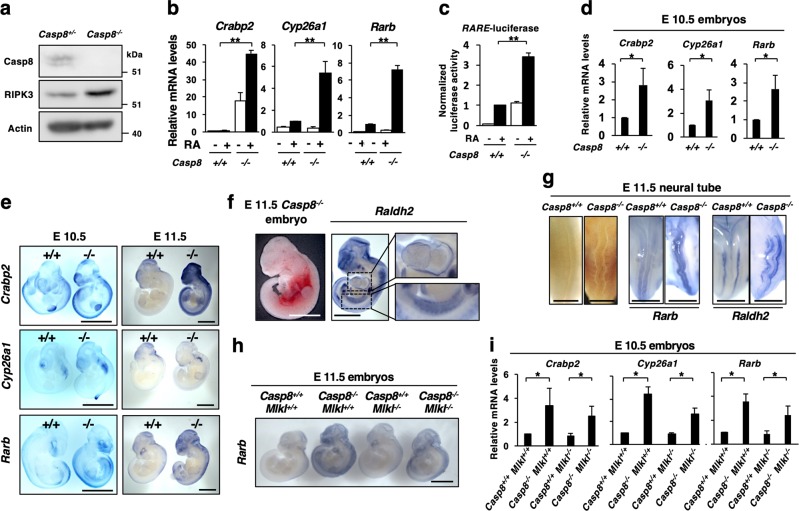
RA signaling was enhanced in *Casp8*^*−/−*^ mouse embryos. **a** Immortalized MEFs derived from Wt (*Casp8*^+/+^) and *Casp8*^−/−^ embryos were subjected to western blot analysis for Caspase-8 and RIPK3. **b** qRT-PCR analysis of RA-induced genes, *Crabp2, Cyp26a1*, and *Rarb*, was carried out in *Casp8*^+/+^ and *Casp8*^−/−^ MEFs after treatment with or without 1 μM RA for 24 h. **c** Dual-luciferase reporter analysis of *RARE* was carried out using RA-treated *Casp8*^+/+^ and *Casp8*^−/−^ MEFs. **d** qRT-PCR analysis of RA-inducible genes, *Crabp2, Cyp26a1*, and *Rarb*, was performed in E10.5 Wt (*Casp8*^+/+^) and *Casp8*^−/−^ littermates (*n* = 10). RNA was extracted from whole embryos. **e** Expressions of RA-inducible genes, *Crabp2, Cyp26a1*, and *Rarb* were analyzed in E10.5 or E11.5 Wt (*Casp8*^+/+^) and *Casp8*^−/−^ littermates by whole-mount in situ hybridization analysis. Scale bars, 2 mm. **f, g** Whole-mount in situ hybridization analysis of *Rarb* and *Raldh2* was carried out using E11.5 *Casp8*^−/−^ embryos (*n* = 5). Scale bars, 2 mm. Views of heart and AGM (**f**), and neural tube (**g**) were shown. Scale bars, 1 mm. **h** Whole-mount in situ hybridization analysis of *Rarb* was carried out using representative E11.5 embryos with the indicated genotypes (*n* = 5). Scale bars, 2 mm. **i** qRT-PCR analysis of RA-inducible genes, *Crabp2, Cyp26a1*, and *Rarb*, was carried out as described in **d**. **p < 0.01 and *p < 0.05

*Casp8*-deficient (*Casp8*^*−/−*^) mouse embryos die at E11.5 with associated abnormal yolk sac vascularization, heart development, and neural tube formation [9, 15]. In mouse embryonic fibroblasts (MEFs) from *Casp8*^*−/−*^ mice (Fig. 8a), RA-induced gene expression as well as *RARE*-dependent transcription of luciferase was enhanced, compared with *Casp8*^*+/+*^ MEFs (Fig. 8b, c). We also detected upregulated transcription of RA-induced genes in *Casp8*^*−/−*^ whole embryos at E10.5 by qRT-PCR and in situ hybridization analyses (Fig. 8d, e). The upregulation was prominent in the embryo at E11.5 specifically in heart, aorta-gonad-mesonephros (AGM) and neural tube, all of which were abnormal in *Casp8*^*−/−*^ embryos (Fig. 8e–g). Thus, RA signaling was enhanced in *Casp8*^*−/−*^ embryos as well as *Casp8* KD cells.

A chemical inhibitor of RA signaling, BMS493 [47], was intraperitoneally injected into the pregnant *Casp8* heterozygous (*Casp8*^*+/−*^) female mice intercrossed with *Casp8*^*+/−*^ male mice (Supplementary Fig. S12a). The upregulated expression of RA-specific target genes in *Casp8*^*−/−*^ embryos was partly but significantly suppressed by treatment with BMS493 (Supplementary Fig. S12b). BMS493-treated *Casp8*-deficient embryos were observed to be viable even at E11.5, and the characteristic abnormal phenotypes of yolk sac, neural tube and heart in *Casp8*-deficient embryos were rescued by injection of BMS493 (Supplementary Fig. S12c–e). Moreover, the enhanced expression of necroptosis-related genes such as *Ripk1*, *Ripk3*, and *Mlkl* in *Casp8*^*−/−*^ embryos were suppressed by BMS493 treatment (Supplementary Fig. S13a). It should be noted, however, that BMS493-treated E12.5 *Casp8*-deficient embryos were not viable (Supplementary Fig. S13b). Thus, chemical inhibition of RA signaling delayed, but did not completely inhibit, embryonic lethality of *Casp8*-deficient embryos.

Embryonic lethality of *Casp8* deficient mice was reported to be completely rescued by knockout of *Mlkl* [17]. In the CRISPR/CAS9-mediated *Mlkl*^*−/−*^ mice, the expression of MLKL protein was completely diminished in both embryos and adult tails (Supplementary Fig. S14a-d). MEFs derived from the *Mlkl*^*−/−*^ embryos were resistant to TNFα-induced necroptosis but not to TNFα-induced apoptosis (Supplementary Fig. S14e). In addition, *Mlkl* KO rescued the embryonic lethality caused by loss of *Casp8* gene (Supplementary Fig. S14f). Then, we analyzed the effect of loss of *Mlkl* expression on the expression of RA-specific target genes in *Casp8*^−/−^ mice. Whole-mount in situ hybridization and qRT-PCR analyses revealed that the enhanced expression of *Crabp2*, *Cyp26a1*, and *Rarb* was observed in *Casp8*^−/−^*Mlkl*^−/−^ embryos (Fig. 8h, i). Taken together, the enhancement of necroptosis, but not enhancement of RA signaling, by loss of *Casp8* expression is essential for embryonic lethality of *Casp8*^*−/−*^ mice, while the augmentation of RA signaling may be involved in the enhancement of necroptosis thorough improvement of RA-induced expression of *Mlkl*, *Ripk1*, and *Ripk3*.

## Discussion

RA-induced necroptosis was observed in EBs derived from *Casp8* KD ES and P19 cells, but not in either *Casp8* KD ES and P19 cells without EB formation. RA-induced necroptosis seems to be induced in only EB or its related early embryo. In addition, the enhanced expressions of *Ripk1*, *Ripk3*, and *Mlkl* were observed not only in RA-treated EBs derived from *Casp8* KD ES cells but also in *Casp8*-deficient E10.5 embryos. The expression level of TNFα also increased in EBs derived from *Casp8* KD ES cells after RA treatment. RA treatment might be able to induce necroptosis in EBs and early embryos through the enhanced expression of these necroptosis-related genes in the absence of the *Casp8* expression. The mechanism that controls the enhanced expression of these genes specifically in EBs or early embryo is a next question to be clarified.

Nuclear translocation of RIPKs was reported in TNFα-induced necroptosis [48, 49]. Our observations indicated that RIPKs translocated into the nucleus and bound to RARα by inhibition of Caspase-8 activity. Recently, RARγ was reported to translocate to cytoplasm and to bind to RIPK1 [50]. Taken together, RARs might have a potential to bind to RIPKs, and therefore RARα and RARγ might be able to bind to RIPK1 and RIPK3 in the nucleus and RIPK1 in the cytoplasm, respectively.

The embryonic lethality of *Casp8*^*−/−*^ embryos is rescued by not only depletion of *Ripk3* [10, 16] but also knock-in of kinase-negative K51A *Ripk3* [51], indicating that kinase activity of RIPK3, which is indispensable for induction of necroptosis, is required for embryonic lethality of *Casp8*^*−/−*^ embryos. Recently, ∆RHIM domain mutant in *Ripk3* gene was reported to rescue the embryonic lethality at E 11.5 of *Fadd*^*−*/*−*^ mice [52]. In addition, *Casp8*^*−/−*^*Mlkl*^*−/−*^ mice were reported to be viable and to mature into fertile adults [17]. In contrast, RHIM domain of RIPK3, but not kinase activity of RIPK3, are required for the enhancement of RA signaling in *Casp8* KD ES cells. All the data indicate that the embryonic lethality of *Casp8*^*−/−*^ mice is due to excess necroptosis [10, 16, 17], but not due to the enhancement of RA-induced differentiation. On the other hand, elimination of TNFR1 from *Casp8*^*−/−*^ embryos was reported to delay embryonic lethality from E11.5 until E16.5 [53], indicating the essential role of TNFα-induced necroptosis in the embryonic lethality of E11.5 *Casp8*^*−/−*^ mice. TNFα was shown to induce necroptosis in RA-treated but not RA-untreated EBs derived from *Casp8* KD ES cells, and the expression level of TNFα was shown to increase after RA treatment in *Casp8* KD ES cells-derived EBs. Taken together, we suppose that *Ripk1*, *Ripk3, Mlkl*, and *TNFα* expressions are enhanced by RA in *Casp8*^*−/−*^ embryos, and their increased expressions might be partly involved in the embryonic lethality of *Casp8*^*−/−*^ mice around E10.5 through enhancing the sensitivity to TNFα-induced necroptosis (Supplementary Fig. S15).

## Supplementary information

Supplementary Figure Legends

Supplementary Tables

Supplementary Fgiure S1

Supplementary Fgiure S2

Supplementary Fgiure S3

Supplementary Fgiure S4

Supplementary Fgiure S5

Supplementary Fgiure S6

Supplementary Fgiure S7

Supplementary Fgiure S8

Supplementary Fgiure S9

Supplementary Fgiure S10

Supplementary Fgiure S11

Supplementary Fgiure S12

Supplementary Fgiure S13

Supplementary Fgiure S14

Supplementary Fgiure S15
