# Supplementary material for: Caspase-8, receptor-interacting protein kinase 1 (RIPK1), and RIPK3 regulate retinoic acid-induced cell differentiation and necroptosis
Source: Cell Death Differ. 2019 Oct 28;27(5):1539–53. doi: 10.1038/s41418-019-0434-2 (PMC7206185; doi:10.1038/s41418-019-0434-2)
Supplement: Supplementary file 1 — Supplementary Figure Legends [file 41418_2019_434_MOESM1_ESM.docx]

**Supplementary Material**

Supplementary Figure Legends

Supplementary Figure S1

Supplementary Figure S2

Supplementary Figure S3

Supplementary Figure S4

Supplementary Figure S5

Supplementary Figure S6

Supplementary Figure S7

Supplementary Figure S8

Supplementary Figure S9

Supplementary Figure S10

Supplementary Figure S11

Supplementary Figure S12

Supplementary Figure S13

Supplementary Figure S14

Supplementary Figure S15

**Supplementary Figure Legends**

**Supplementary Figure S1.** RA signaling in *Casp8* KD ES and P19 cells. **a** Tet-On sh*Casp8* and Tet-On sh*GFP* ES cells were cultured for 4 days with or without 1 µg/ml Dox and then treated with or without 1 nM, 10 n M, 100 nM or 1 µM RA for 24 hours in the presence or absence of Dox. Subsequently, the expression levels of RA-inducible genes, *Crabp2, Hoxb1, Cyp26a1* and *Rarb*, were analyzed by qRT-PCR. **b** Tet-On sh*LacZ* or Tet-On sh*Casp8* #2 P19 cells were cultured for 4 days with or without 1 µg/ml Dox and then treated with or without 1 µM RA for 24 hours. Subsequently, qRT-PCR analysis of *Casp8* and RA-induced genes, *Crabp2*, *Hoxb1* and *Cyp26a1*, was carried out. **c** qRT-PCR analysis of RA receptors, *Rara*, *Rarg* and *Rxra*, was carried out using Tet-On sh*Casp8* and Tet-On sh*GFP* ES cells after treatment with or without 1 μM RA for 24 hours in the presence or absence of 1 µg/ml Dox. **p < 0.01 and n.s.d. (no significant difference: p > 0.95).

**Supplementary Figure 2.** RA-induced differentiation of *Casp8* KD P19 and *Fadd* KD ES cells. **a** Tet-On sh*Casp8* and Tet-On sh*LacZ* P19 cells were cultured with or without 1 µg/ml Dox for 4 days and then treated with 1 μM RA for 24 hours in the presence or absence of Dox. Subsequently, western blot analysis with anti-Caspase-8 antibody was performed. Actin was detected as a control. **b** qRT-PCR analysis of *Nestin* and *Tuj1* was carried out using Tet-On sh*Casp8* or Tet-On sh*LacZ* P19 cells at the indicated times after formation of EBs in the presence or absence of 1 µg/ml Dox. EBs were cultured with or without 1 μM RA after 2 days formation of EBs. **c** qRT-PCR analysis of RA-inducible genes, *Crabp2, Hoxb1* and *Cyp26a1*, was carried out using Tet-On sh*Casp8* P19 cells after treatment with or without 1 µM RA for 24 hours in the presence or absence of 1 µg/ml Dox. **d** qRT-PCR analysis of *Fadd*, *Nestin* and *Tuj1* was carried out in Tet-On sh*GFP* and Tet-On sh*Fadd* ES cells at the indicated days after formation of EBs in the presence or absence of 1 µg/ml Dox. EBs were cultured with or without 1 μM RA after 2 days formation of EBs. **e** Tet-On sh*Fadd* and Tet-On sh*GFP* ES cells were cultured for 4 days with or without 1 μg/ml Dox and then treated with or without 1 μM RA for 24 hours in the presence or absence of Dox. Subsequently, qRT-PCR analysis of RA-induced genes, *Crabp2*, *Hoxb1* and *Cyp26a1*, was performed. **p < 0.01 and *p < 0.05.

**Supplementary Figure S3.** RA-induced differentiation of *Casp8* KD human SK-N-SH and HL60 cells. **a** Western blot analysis with anti-Caspase-8 antibody was carried out using human neuroblastoma-derived SK-N-SH cells stably expressing sh*CASP8* or sh*LacZ*. Actin was detected as a control. **b** Neuronal differentiation of SK-N-SH cells expressing sh*CASP8* or sh*LacZ* was induced by treatment with 1 μM RA for 3 days, and observed by phase-contrast microscopy. Scale bars, 200 μm. **c** qRT-PCR analysis of *TUJ1* and RA-induced genes, *CRABP2*, *CYP26A1,* and *RARB*, was carried out using SK-N-SH cells expressing sh*LacZ* or sh*CASP8* after treatment with or without 1 μM RA for 24 hours. **d** Human promyelocytic leukemia-derived HL60 cells stably expressing sh*LacZ* or sh*CASP8* were monitored for Caspase-8 depletion by western blot analysis with an anti-Caspase-8 antibody. Actin was detected as a control. Molecular weight markers are indicated (kDa). **e** qRT-PCR analysis of *Rarb* and *CD11b* expression was carried out using HL60 cells expressing sh*LacZ* or sh*CASP8* before and after treatment with or without 1 μM RA for 3 days.. **f** HL60 cells expressing sh*LacZ* or sh*CASP8* were treated with or without 1 μM RA for 3 days, stained with an FITC-conjugated anti-CD11b antibody and subjected to flow cytometric analysis. **p < 0.01 and *p < 0.05.

**Supplementary Figure S4.** Protease activity of procaspase-8 regulates the evident activation of RA signaling. **a** Western blot analysis with an anti-Caspase-8 antibody was carried out using Tet-On sh*Casp8* P19 cells expressing the following *Casp8* cDNA constructs: Wt, a protease-inactive mutant (CS) with a C362S mutation in the protease domain, or a mutant in putative cleavage sites (DE) with seven Asp to Glu mutations which were expected to be cleaved by Caspase-8 itself and other caspases. All the *Casp8* cDNA constructs carried silent mutations in the sh*Casp8* target sequence. After treatment with 1 µg/ml Dox for 4 days, western blot analysis was performed. Actin was detected as a control. Molecular weight markers are indicated (kDa). **b** Tet-On sh*Casp8* P19 cells defined in (**a**) were cultured for 4 days with or without 1 µg/ml Dox and then treated with or without 1 µM RA for 24 hours in the presence or absence of Dox. Subsequently, the expression levels of RA-inducible genes, *Crabp2, Hoxb1,* and *Cyp26a1*, were analyzed by qRT-PCR. Vector, an empty vector. **p < 0.01 and *p < 0.05.

**Supplementary Figure S5.** Effector caspases are not involved in the activation of RA signaling in the absence of Caspase-8. **a** Tet-On sh*LacZ* P19 cells or sh*Casp7* expressing Tet-On sh*Casp3* P19 cells were cultured for 4 days with or without 1 µg/ml Dox and then treated with or without 1 µM RA for 24 hours in the presence or absence of Dox. Subsequently, qRT-PCR analysis of *Casp3*, *Casp7*, or RA-induced genes, *Crabp2*, *Hoxb1* and *Cyp26a1*, was carried out. **b** Tet-On sh*Casp8* P19 cells expressing sh*LacZ* or both sh*Casp3* and sh*Casp7* were cultured for 4 days with or without 1 µg/ml Dox and then treated with or without 1 µM RA for 24 hours in the presence or absence of Dox. Subsequently, qRT-PCR analysis of *Casp3*, *Casp7, Casp8* or RA-induced genes, *Crabp2*, *Hoxb1* and *Cyp26a1*, was carried out. **p < 0.01 and n.s.d. (no significant difference: p > 0.95).

**Supplementary Figure S6.** Characterization of RA-induced cell death in EBs. **a** Dox (1 μg/ml)-treated or -untreated Tet-On shLacZ or Tet-On sh*Casp8 #2* ES cells were analyzed by phase-contrast microscopy after 4 days formation of EBs. EBs were treated with or without 1 μM RA for last 2 days. **b** Western blot analysis with an anti-caspase-8 antibody was performed using Tet-On sh*Casp8* ES cells expressing Wt, CS and DE *Casp8* cDNA constructs at the indicated times after formation of EBs in the presence or absence of 1 µg/ml Dox. EBs were cultured with or without 1 μM RA after 2 days formation of EBs. Actin was detected as a control. Molecular weight markers are indicated (kDa). **c** Cell death was quantified by LDH release assay after 4 days of formation of EBs derived from Tet-On sh*Casp8* ES cells expressing WT, CS and DE *Casp8* cDNA constructs. EBs were treated with or without 1 μM RA for last 2 days. **d** Cell death was quantified by LDH release assay after 4 days formation of EBs derived from Tet-On sh*Casp8* ES cells. EBs were treated with or without 1 μM RA in the present of DMSO or 30 µM zVAD-fmk for last 2 days. **p < 0.01.

**Supplementary Figure S7.** Expression of TNF*α* and IFNs in EBs during RA-induced necroptosis. qRT-PCR analysis of *TNFα,* *IFNα*, *IFNβ* and *IFNγ* was performed using Tet-On sh*Casp8* ES cells after 4 days formation of EBs in the presence or absence of 1 μg/ml Dox. EBs were cultured with or without 1 μM RA for last 2 days. Casp8 KD ES cells or EBs were treated with 1 µg/ml Dox throughout the experiments. *p < 0.05 and n.s.d. (no significant difference: p > 0.95).

**Supplementary Figure S8.** The ligand-binding domain (LBD) of RARα directly interacted with the protein kinase domain (PKD) of RIPK1 and RIPK3. **a** A graphical overview of 3xFlag-tagged full-length human RARα (Wt) and its various deletion mutants. The numbers of amino acid residues in RARα are indicated. N, N-terminal domain; IR, intermediate region; DBD, DNA-binding domain; and LBD, ligand binding domain. **b** Lysates of HEK293T cells transiently expressing EGFP-RIPK3 and various deletion mutants of 3xFlag-RARα were subjected to immunoprecipitation (IP) with anti-Flag or anti-GFP antibodies, and analyzed by western blotting with anti-Flag or anti-GFP antibodies. Total cell lysates (Lysate) were also analyzed. Molecular weight markers are indicated (kDa). **c** A graphical overview of 3xFlag-tagged full-length mouse RIPK3 (Wt) and its various deletion mutants. Relevant numbers of amino acid residues in RIPK3 are indicated. RHIM, RIP homotypic interaction motif domain; IR, intermediate region; and PKD, protein kinase domain. **d** HEK293T cells transiently expressing EGFP-RARα or various deletion mutants of 3xFlag-RIPK3 were subjected to immunoprecipitation (IP) with anti-Flag or anti-GFP antibodies, and analyzed by western blotting with anti-Flag or anti-GFP antibodies. Total cell lysates (Lysate) were also analyzed. **e, f** *In vitro* binding assay of purified HA-RARα to Flag-RIPK3 (**e**) or Flag-RIPK1 (**f**) was carried out by immunoprecipitation (IP) with anti-Flag antibody after incubation of purified proteins. The immunoprecipitates with anti-Flag antibody were analyzed with anti-Flag, anti-HA, and anti-RIPK1 (**e**) or anti-RIPK3 (**f**) antibodies. Purified recombinant RIPK1 and RIPK3 do not contain endogenous RIPK3 and RIPK1, respectively.

**Supplementary Figure S9.** RHIM domains and their mutants of RIPK1 and RIPK3. Graphical overviews of EGFP-tagged full-length mouse RIPK1, EGFP-tagged RHIM domain of mouse RIPK1 containing NLS, and EGFP-tagged RHIM domain of mouse RIPK1with AAAA mutation; and mCherry-tagged full-length mouse RIPK3, mCherry-tagged RHIM domain of mouse RIPK3 containing NLS, and mCherry-tagged RHIM domain of mouse RIPK3 with an AAAA mutation. In mouse RIPK1 and RIPK3 AAAA mutants, amino acid sequences IQIG in RHIM domain of Wt RIPK1 and VQIG in RHIM domain of Wt RIPK3, respectively, were replaced by amino acids AAAA. DD, death domain

**Supplementary Figure S10.**  RHIM domains of mouse RIPK1 and RIPK3 are important for not only forming a nuclear complex with RARα but also *Casp8* knockdown-induced enhancement of RA signaling. **a, b** P19 cells were transfected with an expression vector encoding EGFP-RHIM of RIPK1 (RHIM of RIPK1) or EGFP-RHIM of RIPK1 with the AAAA mutation (AAAA RHIM of RIPK1) together with (**b**) or without (**a**) that encoding mCherry-RARα; or an expression vector encoding mCherry-RHIM of RIPK3 (RHIM of RIPK3) or mCherry-RHIM of RIPK3 with the AAAA mutation (AAAA RHIM of RIPK3) together with (**b**) or without (**a**) that encoding EGFP-RARα. Cells were cultured for 48 hours, and subcellular localization of these proteins was analyzed by confocal fluorescence microscopy after staining with DAPI. Scale bars, 20 μm. **c** Lysates of HEK293Tcells transiently expressing EGFP-RARα together with or without 3xFlag-Wt RIPK3 or 3xFlag-RIPK3 with the AAAA mutation in its RHIM domain (3xFlag-AAAA RIPK3) were subjected to immunoprecipitation with anti-Flag antibody or anti-GFP antibody, and analyzed by western blotting with anti-Flag antibody or anti-GFP antibody. Total cell lysates (input) were also analyzed. Molecular weight markers are indicated (kDa). **d, e** Tet-On sh*Casp8* P19 cells expressing sh*LacZ* or sh*Ripk3* were infected with lentiviral vectors carrying 3xFlag-Wt *Ripk3* or *Ripk3* with the AAAA mutation (AAAA RIPK3), cultured with or without 1 μg/ml Dox for 5 days, and then treated with or without 1 μM RA for 24 hours in the presence or absence of Dox. Subsequently, western blot analysis for RIPK3 and Actin (**d**) and qRT-PCR analysis for RA-inducible genes (**e**) were carried out. Vector, an empty vector.

**Supplementary Figure S11.** RXRα, TDG, p300 and CBP are required for the marked enhancement of RA signaling in the absence of *Casp8* expression. **a** Tet-On sh*Casp8* P19 cells expressing sh*LacZ* or sh*Rxra* were subjected to qRT-PCR analysis for *Rxra*. **b** P19 cells defined in (**a**) were cultured for 4 days with or without 1 µg/ml Dox and then treated with or without 1 µM RA for 24 hours. Subsequently, qRT-PCR analysis of RA-induced genes, *Crabp2*, *Hoxb1* and *Cyp26a1*, was carried out. **c** The expression levels of *Tdg* were analyzed by qRT-PCR using Tet-On sh*Casp8* P19 cells expressing sh*LacZ* or sh*Tgd*. Two shRNAs targeting different nucleotide sequences in *Tdg* (sh*Tdg* #1 and sh*Tdg* #2) were used. **d** P19 cells defined in (**c**) were cultured for 4 days with or without 1 µg/ml Dox and then treated with or without 1 µM RA for 24 hours. Subsequently, qRT-PCR analysis of RA-induced genes, *Crabp2*, *Hoxb1* and *Cyp26a1*, was carried out. **e** Tet-On sh*Casp8* P19 cells expressing sh*LacZ* or sh*Tdg* #1 together with or without Wt *Tdg* were subjected to qRT-PCR analysis for *Tdg*. **f** P19 cells defined in (**e**) were cultured with or without 1 µM RA for 24 hours in the presence or absence of 1 µg/ml Dox, and then subjected to qRT-PCR analysis for RA-inducible genes. Vector, an empty vector. **g** Tet-On sh*Casp8* P19 cells expressing sh*LacZ* or sh*p300*, and sh*LacZ* or sh*Cbp* were subjected to qRT-PCR analysis for *p300* and *Cbp*, respectively. **h** P19 cells defined in (**g**) were cultured for 4 days with or without 1 µg/ml Dox and then treated with or without 1 µM RA for 24 hours. Subsequently, qRT-PCR analysis of RA-induced genes, *Crabp2*, *Hoxb1* and *Cyp26a1*, was carried out. **p < 0.01 and *p < 0.05.

**Supplementary Figure S12.** Marked enhancement of RA signaling in the absence of Caspase-8 was correlated with the abnormality of *Casp8^-/-^* embryos. **a** Experimental design of intra-peritoneal injection of BMS493 into pregnant *Casp8*^+/-^ mice. **b** Whole-mount *in situ* hybridization analysis of RA-inducible genes, *Crabp2* and *Rarb*, was carried out for E11.5 Wt (*Casp8*^+/+^) and *Casp8*^-/-^ littermates, and E11.5 *Casp8*^-/-^ embryos from BMS493-treated pregnant *Casp8*^+/-^ mice. Scale bars, 2 mm. **c** Views of yolk sac of E11.5 *Casp8*^-/-^ embryos from the pregnant *Casp8*^+/-^ mice treated with or without BMS493. Scale bars, 1 mm. **d** Views of neural tube of E11.5 *Casp8*^+/+^ and *Casp8*^-/-^ embryos, and E11.5 *Casp8*^-/-^ embryo from BMS493-treated pregnant *Casp8*^+/-^ mice. Scale bars, 1 mm. **e** Quantification of viability and abnormalities of E11.5 *Casp8*^-/-^ embryos from the pregnant *Casp8*^+/-^ mice treated with or without BMS493. Typical images of healthy, alive but abnormal, and dead E11.5 *Casp8*^-/-^ embryos were indicated. Scale bars, 2 mm.

**Supplementary Figure S13.** Inhibition of RA signaling in *Casp8^-/-^* embryos suppresses the enhanced expression of necroptosis-related genes and delays embryonic lethality. **a** Total RNA was extracted from E 10.5 embryos from pregnant *Casp8^+/-^* mice intra-peritoneally injected with BMS493. Then, qRT-PCR analysis of *Ripk3, Ripk1* and *Mlkl* was performed. **b** Representative views of *Casp8*^-/-^, BMS493-treated *Casp8*^+/+^ and BMS493-treated *Casp8*^-/-^ E12.5 embryos are shown. Scale bars, 2 mm. **p < 0.01 and *p < 0.05.

**Supplementary Figure S14.** Generation and analyses of CRISPR/CAS9-mediated *Mlkl^-/-^* mice. **a** CRISPR/CAS9-mediated knockout of *Mlkl* gene. The sequence targeted by the used small guide RNA is indicated. The protospacer-adjacent motif (PAM) sequence is depicted in red. **b** Sequences of the wild-type *Mlkl* locus and of the targeted *Mlkl* locus in CRISPR/CAS9-mediated *Mlkl^-/-^* mice. CRISPR/CAS9-mediated *Mlkl^-/-^* mice carry one base pair insertion in *Mlkl* gene. **c** Primary MEFs derived from *Mlkl*^+/+^, *Mlkl*^+/-^, or *Mlkl*^-/-^ embryos were subjected to western blot analysis with anti-MLKL antibody. Molecular weight markers are indicated (kDa). **d** The expression of MLKL protein in tails of the adult *Mlkl*^+/+^*, Mlkl*^+/-^ and *Mlkl*^-/-^ mice was analyzed by western blotting with anti-MLKL antibody. NIH3T3 cell lysate was used as control. **e** Primary MEFs derived from *Mlkl*^+/+^ or *Mlkl*^-/-^ embryos were stimulated with DMSO, 10 ng/ml TNFα (T), 0.1 µg/ml Cycloheximide (CHX)(C), 30 µM zVAD-fmk (Z), 10 ng/ml TNFα plus 0.1 µg/ml CHX (TC), or 10 ng/ml TNFα plus 0.1 µg/ml CHX and 30 µM zVAD-fmk (TCZ) for 2 days, and stained with Propidine iodide (PI). Then, cells were analyzed by phase-contrast microscopy and fluorescence microscopy, and representative data were shown. Scale bars, 10 µm. **f** Microscopic examination of embryos with the indicated genotypes. The embryos were isolated from pregnant mice at E10.5, E11.5, E13.5 and E15.5. Scale bars, 5 mm.

**Supplementary Figure S15.** A model of the RA signaling in the presence and absence of Caspase-8. Brown directional lines indicate signaling pathways enhanced or induced by the absence of Caspase-8 activity.
