# Supplementary material for: Caspase-8, receptor-interacting protein kinase 1 (RIPK1), and RIPK3 regulate retinoic acid-induced cell differentiation and necroptosis
Source: Cell Death Differ. 2019 Oct 28;27(5):1539–53. doi: 10.1038/s41418-019-0434-2 (PMC7206185; doi:10.1038/s41418-019-0434-2)
Supplement: Supplementary file 2 — Supplementary Tables [file 41418_2019_434_MOESM2_ESM.docx]

**Supplementary Material**

Supplementary Tables

Supplementary Table S1

Supplementary Table S2

Supplementary Table S3

Supplementary Table S4

**Supplementary Tables**

**Supplementary Table S1 Target sequences of shRNA**

| Target Gene | Accession number | Sequence (5' to 3') |
| --- | --- | --- |
| Casp8 (mouse) | NM_009812.2 | GGAAGATCGAGGATTATGAAA |
| Casp8 #2 (mouse) | NM_009812.2 | GAGAGGAGATGGTGAGAGAGCT |
| Casp7 (mouse) | NM_007611.2 | GACCTGATTACGGAA |
| Casp3 (mouse) | NM_009810.3 | CACTTTCCACGCAAAGAAA |
| Fadd (mouse) | NM_010175.5 | GCGAGCGCGTGAGCAAACGAA |
| Ripk3 (mouse) | NM_019955.2 | TATGGTTATTCTTCGTAATGA |
| Ripk1 (mouse) | NM_009068.3 | CCTGAATGACATCAATGCAAA |
| Mlkl (mouse) #1 | NM_029005.2 | TCCCAACATCTTGCGTATATT |
| Mlkl (mouse) #2 | NM_029005.2 | AGATCCAGTTCAACGATATAT |
| Rxra (mouse) | NM_011305.3 | CCTGTTCAACCCTGACTCTAA |
| Tdg (mouse) #1 | NM_011561.2 | AAATGTCAGGAAGAGTCTTGG |
| Tdg (mouse) #2 | NM_011561.2 | TTAACAGCCATCTTCTTTGCG |
| p300 (mouse) | NM_177821.6 | CCAACAGGAATGACTACCAAT |
| Rarg (mouse) | NM_011244.4 | TGCTTGTCTGGACATCCTAA |
| Cbp (mouse) | NM_001025432.1 | TAACTCTGGCCATAGCTTAAT |
| CASP8 (human) | NM_001228.4 | GAATCACAGACTTTGGACAAA |
| GFP |  | AAGCAGCACGACTTCTTCAAG |
| LacZ | V00296.1 | AAGGCCAGACGCGAATTAT |

**Supplementary Table S2 Primer sequences for qRT-PCR**

| Target Gene | Accession number | Forward primer sequence (5' to 3') | Reverse primer sequence (5' to 3') |
| --- | --- | --- | --- |
| Casp8 (mouse) | NM_009812.2 | CTAGACTGCAACCGA GAGG | GCAGGCTCAAGTCATCTTCC |
| Casp7 (mouse) | NM_007611.2 | CCAGAAGATGGGTAAATGCATCA | ACGTCCATACCTGTCGCTTTG |
| Casp3 (mouse) | NM_009810.3 | CAAAGGACGGGTCGTGGTT | GCGCGTACAGCTTCAGCAT |
| Ripk3 (mouse) | NM_019955.2 | CACATACTTTACCCTTCAGA | TCAGAACAGTTGTTGAAGAC |
| Ripk1 (mouse) | NM_009068.3 | CGTGAGAATATTAAGAGTGC | TGTACCTGTAGTTCCAAATC |
| Mlkl (mouse) | NM_029005.2 | GACCAAACTGAAGACAAGTA | CTCACTATTCCAACACTTTC |
| Crabp2 (mouse) | NM_007759.2 | GAAATGGGAGAGTGGAAACA | GCTCTCCATCATTGGTCAG |
| Hoxb1 (mouse) | NM_008266.5 | GTCTGCTCAGTTCCGTAT | TATTATTCTGTGGGTCAGTC |
| Cyp26a1 (mouse) | NM_007811.2 | GACGTCACTGATCACTTACC | GACATCACTGATCACTTACC |
| Rarb (mouse) | NM_001289761.1 | AACTCAGATGCACAATGCTG | TTTGTTGGTTCCTCAAGGTC |
| Rara (mouse) | NM_009024.2 | AGTTCCGAAGAGATAGTACC | CGTATACACCATGTTCTTCT |
| Rarg (mouse) | NM_011244.4 | CAGGACACTATGACATTCTC | GAGGCAGATAGCACTAAGTA |
| Rxra (mouse) | NM_011305.3 | CTCCATAGCTGTGAAAGAT | TTAGACACCAGCTCTGTTAG |
| Raldh2 (mouse) | NM_009022.4 | CAAGCTCTCCATACTGTTAC | ACATTCTATACTGTGGGTTG |
| p300 (mouse) | NM_177821.6 | TGGACTACCCTATCAAGTAA | CATAGGACTAGCACTCATGT |
| Cbp (mouse) | NM_001025432.1 | AAGCTAAAGAGGAAGAAGAG | TAGAGTGCTTCTAGAGTTGG |
| Tdg (mouse) | NM_172552.3 | CGCTTCAACGGCGTCTCT | TTGAAGGTCAAAATGTCAGGAAG |
| Tuj1 (mouse) | NM_023279.2 | GCTCAAAATGTCATCCACCT | CTCGGTGAACTCCATCTCAT |
| Nestin (mouse) | NM_016701.3 | GGTCTGAGTCTGCTTCCTTG | CAGCCTGCTCTAGTCCATTC |
| Oct3/4 (mouse) | NM_013633.3 | CCGACAACAATGAGAACCTT | ACATGGTCTCCAGACTCCAC |
| TNFα(mouse) | NM_013693.3 | CACAAGATGCTGGGACAGTGA | TCCTTGATGGTGGTGCATGA |
| IFNα(mouse) | NM_010502.2 | ACCTCCACCAGCAGCTCAAT | CCCCACCTGCTGCATCAG |
| IFNβ(mouse) | NM_010510.1 | CGGACTTCAAGATCCCTATGGA | TGGCAAAGGCAGTGTAACTCTTC |
| IFNγ(mouse) | NM_008337.4 | TTGGCTTTGCAGCTCTTCCT | TGACTGTGCCCGTGGCAGTA |
| CYP26A1 (human) | NM_000783.3 | CACATCTCTGATCACTTACC | GAAGGGTCTCCTTAATAACA |
| RARB (human) | NM_001290276.1 | CCGAGATAAGAACTGTGTTA | ACTCAGCTGTCATTTCATAG |
| TUJ1 (human) | NM_001197181.1 | CAAGTTCTGGGAAGTCAT | CAAGTTCTGGGAAGTCAT |
| CD11b (human) | NM_001145808.1 | TCTCTTTGATGCAGTACTCT | GATGTTAAACAGCTCTCGTA |
| GAPDH (human) | NM_002046.5 | TCCACCACCCTGTTGCTGTA | ACCACAGTCCATGCCATCAC |

**Supplementary Table S3 Primer sequences for Chip assay**

| Target gene | Forward primer sequence (5' to 3') | Reverse primer sequence (5' to 3') |
| --- | --- | --- |
| Rarb RARE | CTCTGGCTGTCTGCTTTTGC | CAGCTCACTTCCTACTACTTC |
| Cyp26a1 RARE | TTTTGGGCAGCGCCTCGAGG | CATCTGCAAGGTTTCCCCAA |
| Gapdh promoter | TACTCGCGCTTTACGGG | TGGAACAGGGAGGAGCAGAGAGCA |

**Supplementary Table S4 Target sequence of CRISPR/CAS9 system**

| Target Gene | Accession number | Sequence (5' to 3') |
| --- | --- | --- |
| Mlkl (mouse) | NM_001310613.1 | AAGGAACATCTTGGACCTCCG |
