# Supplementary figures and images for: Caspase-8, receptor-interacting protein kinase 1 (RIPK1), and RIPK3 regulate retinoic acid-induced cell differentiation and necroptosis
Source: Cell Death Differ. 2019 Oct 28;27(5):1539–53. doi: 10.1038/s41418-019-0434-2 (PMC7206185; doi:10.1038/s41418-019-0434-2)

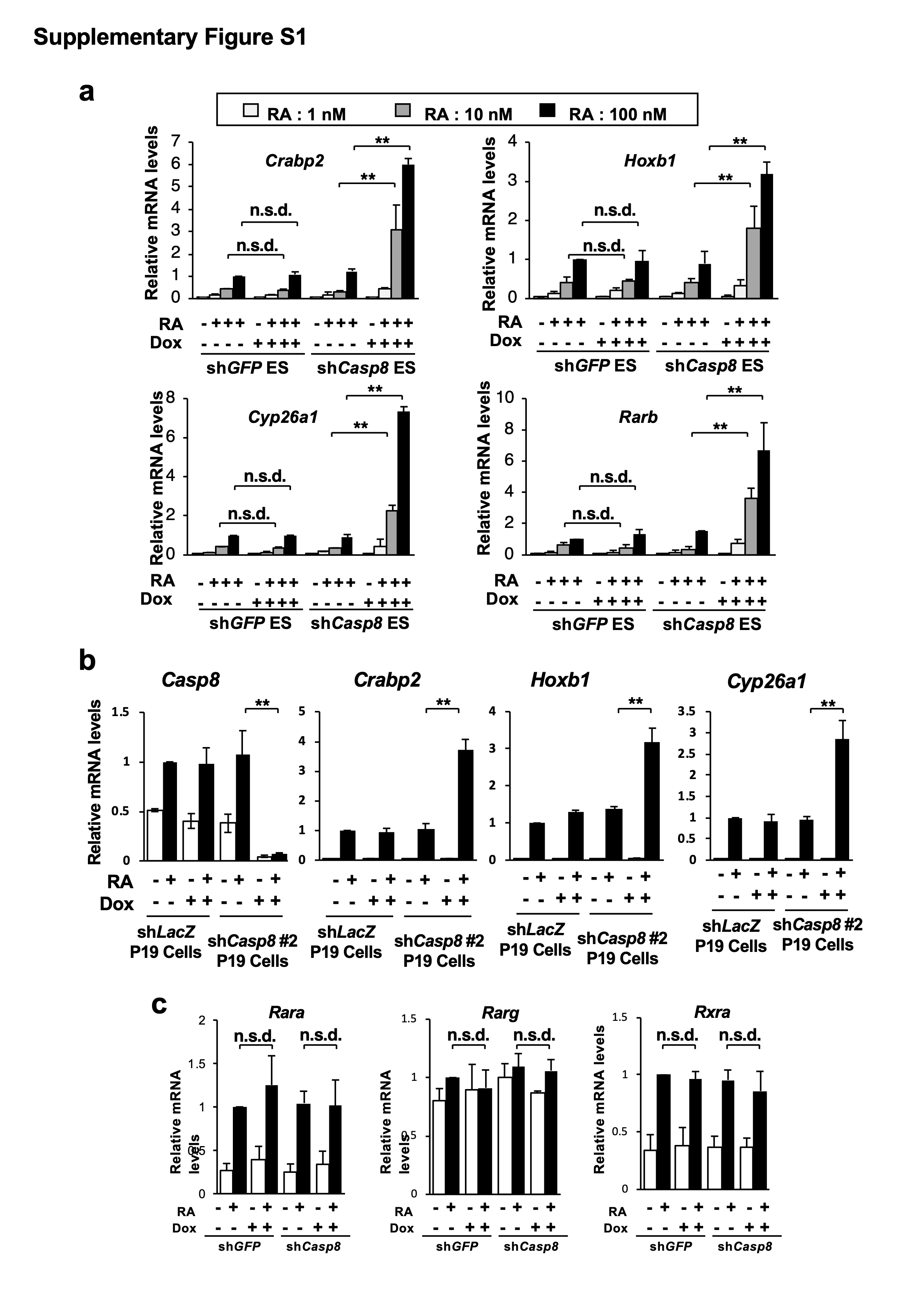

Supplement: Supplementary file 3 — Supplementary Fgiure S1 [file 41418_2019_434_MOESM3_ESM.tif]

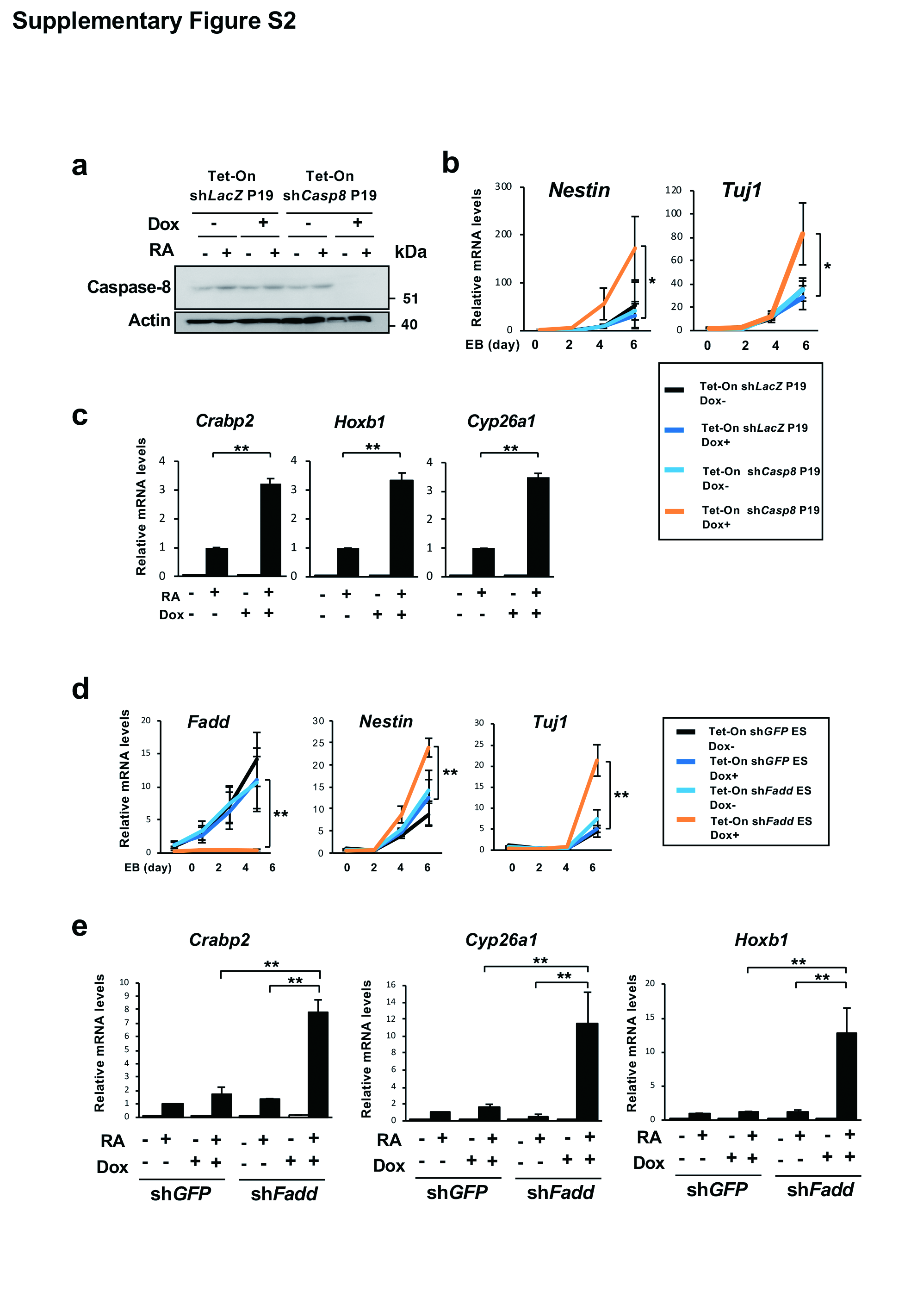

Supplement: Supplementary file 4 — Supplementary Fgiure S2 [file 41418_2019_434_MOESM4_ESM.tif]

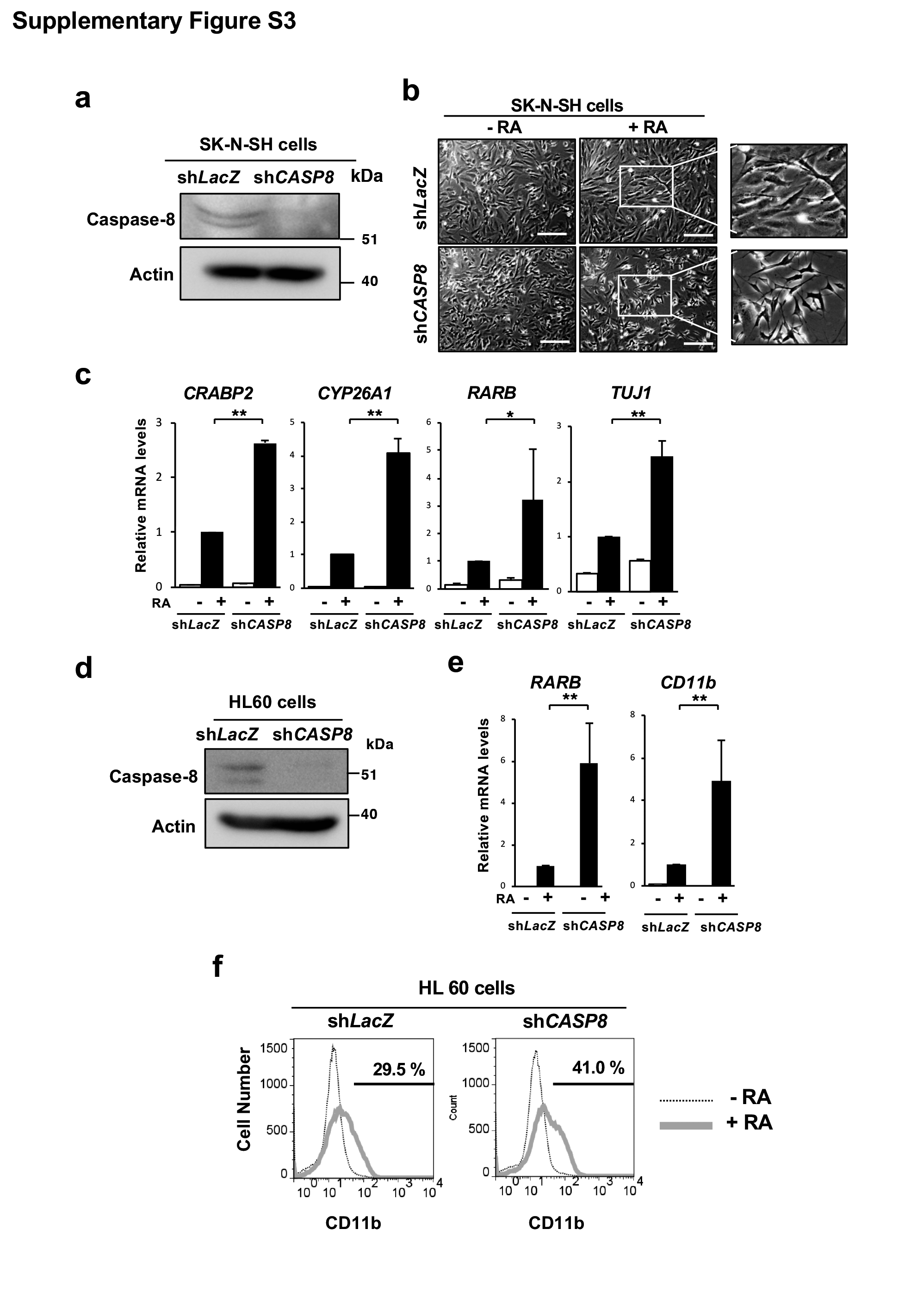

Supplement: Supplementary file 5 — Supplementary Fgiure S3 [file 41418_2019_434_MOESM5_ESM.tif]

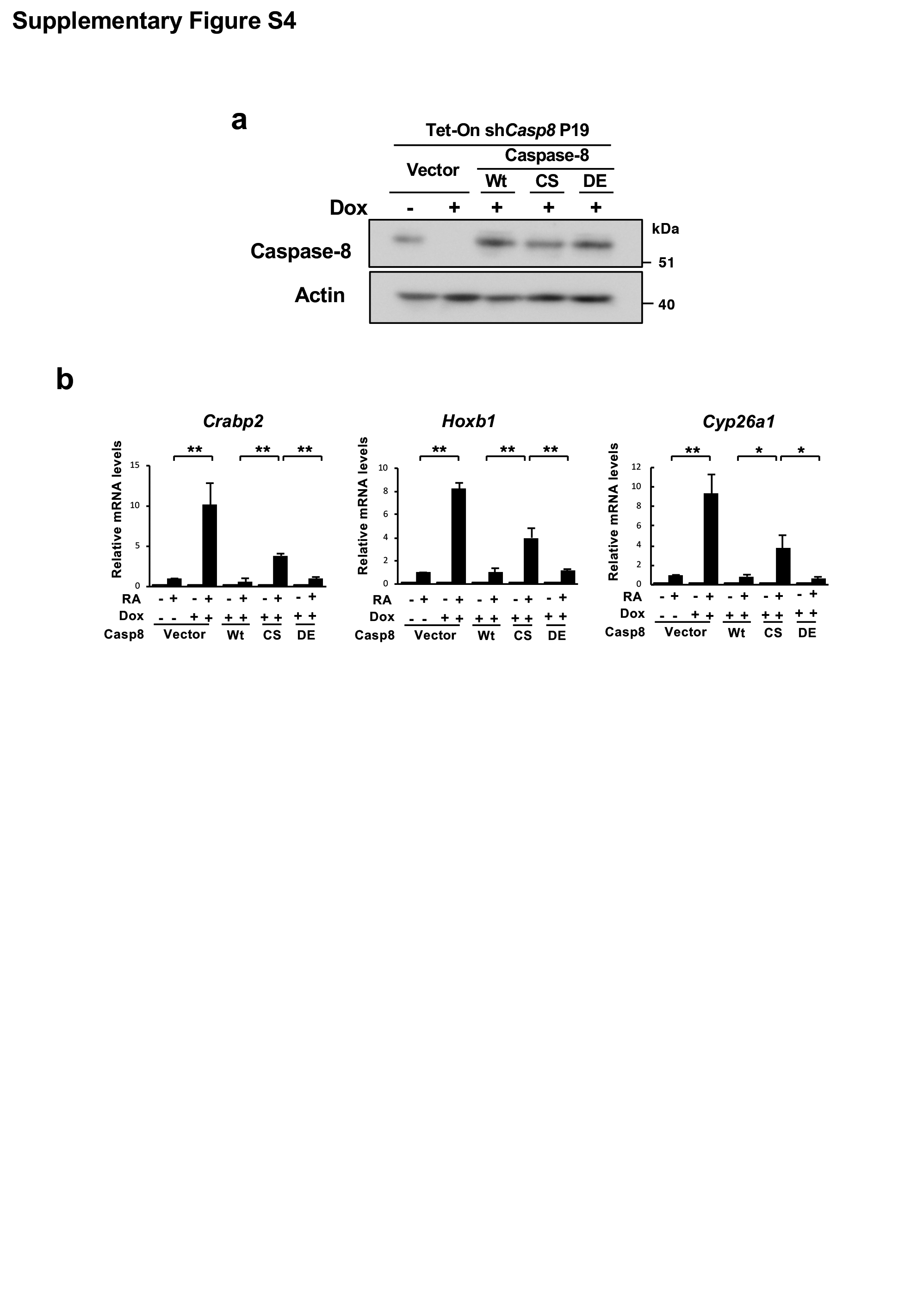

Supplement: Supplementary file 6 — Supplementary Fgiure S4 [file 41418_2019_434_MOESM6_ESM.tif]

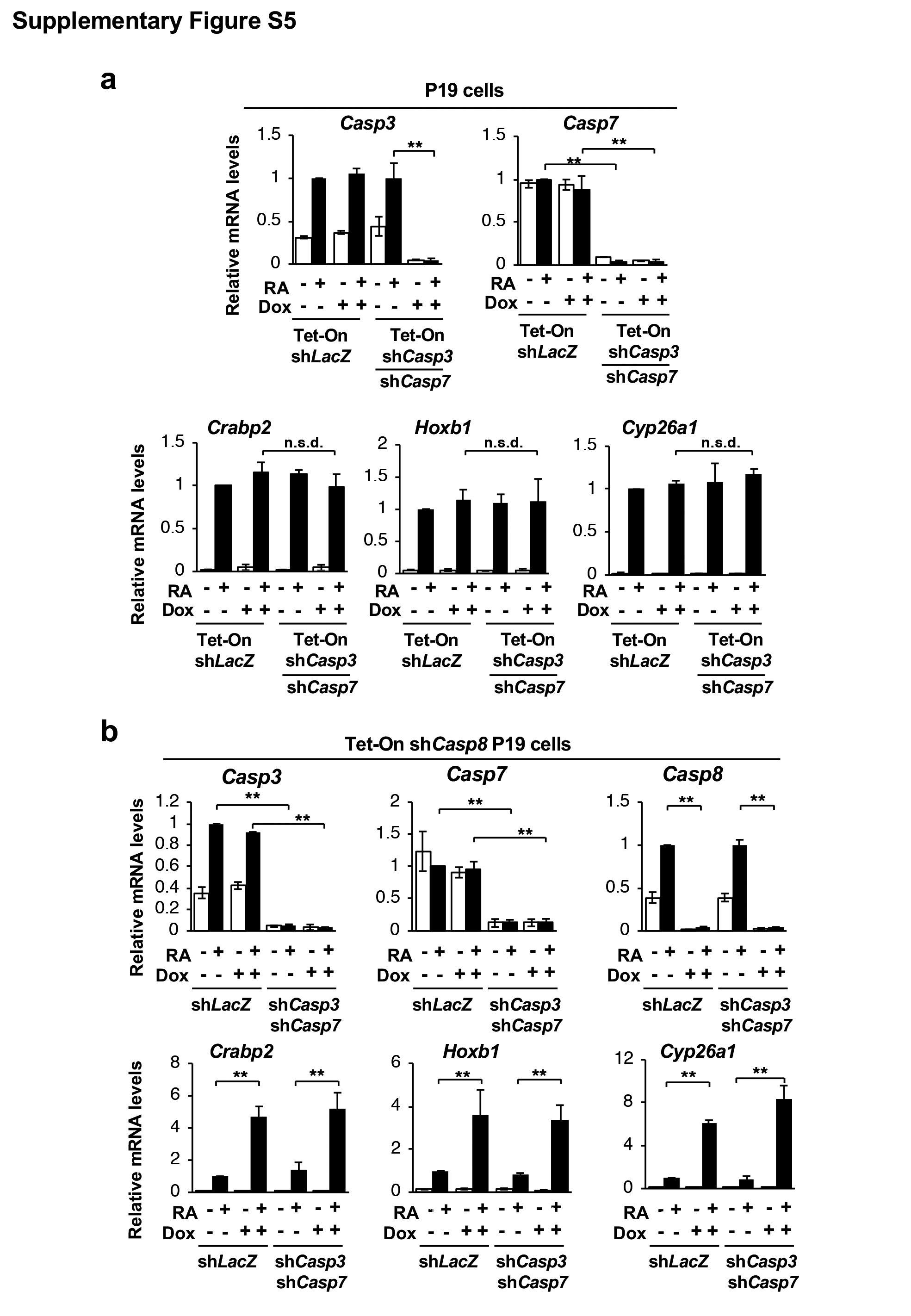

Supplement: Supplementary file 7 — Supplementary Fgiure S5 [file 41418_2019_434_MOESM7_ESM.tif]

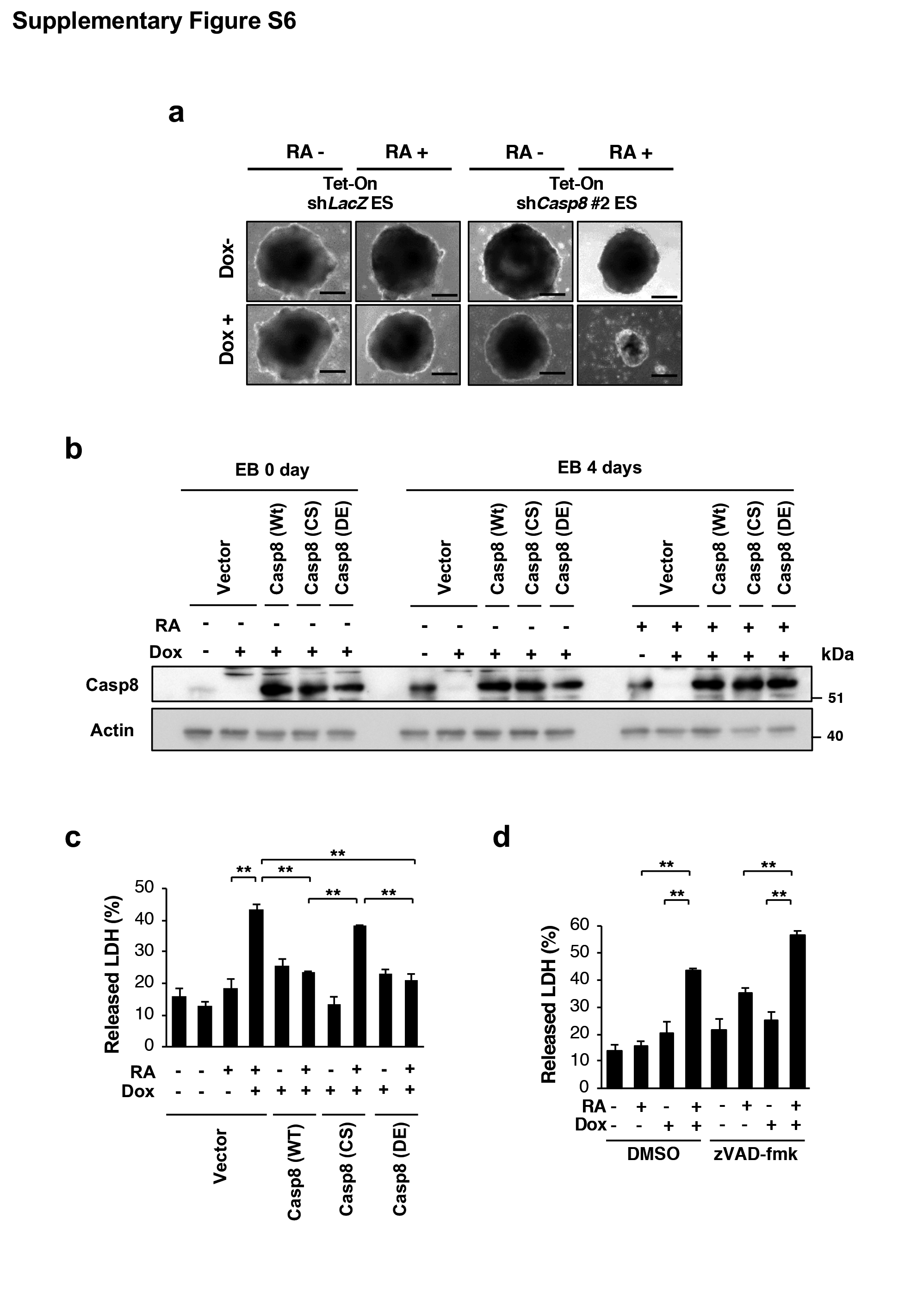

Supplement: Supplementary file 8 — Supplementary Fgiure S6 [file 41418_2019_434_MOESM8_ESM.tif]

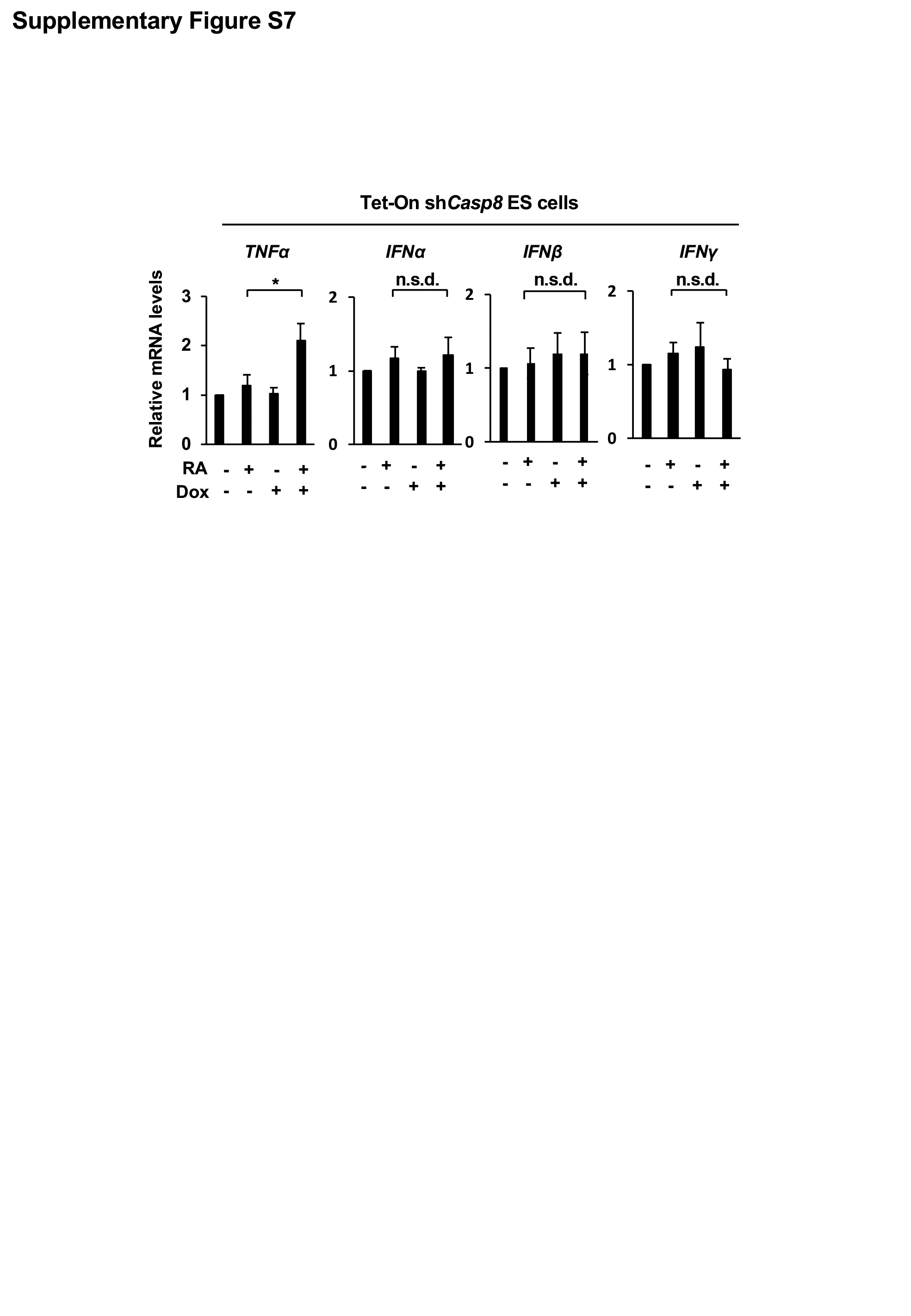

Supplement: Supplementary file 9 — Supplementary Fgiure S7 [file 41418_2019_434_MOESM9_ESM.tif]

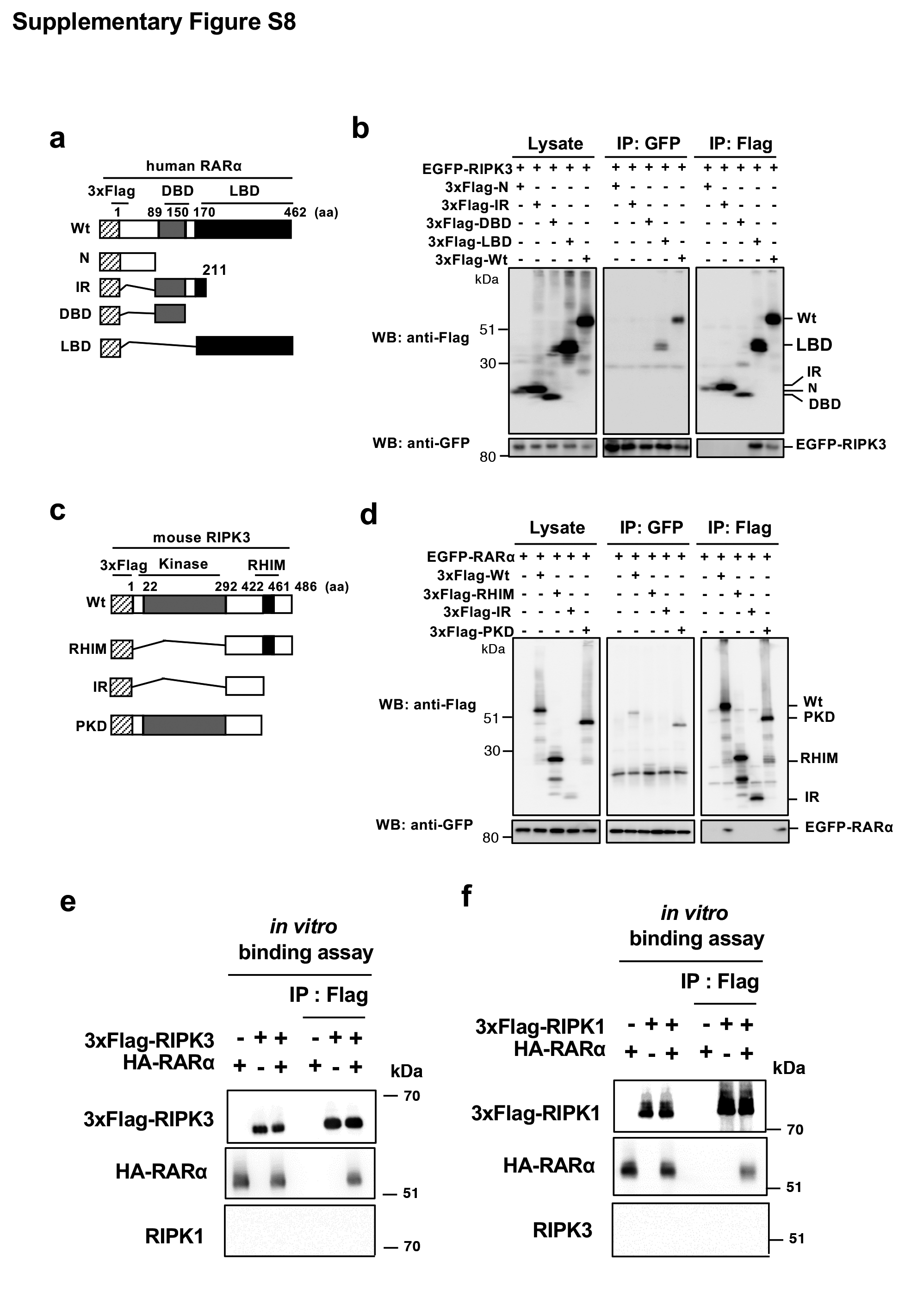

Supplement: Supplementary file 10 — Supplementary Fgiure S8 [file 41418_2019_434_MOESM10_ESM.tif]

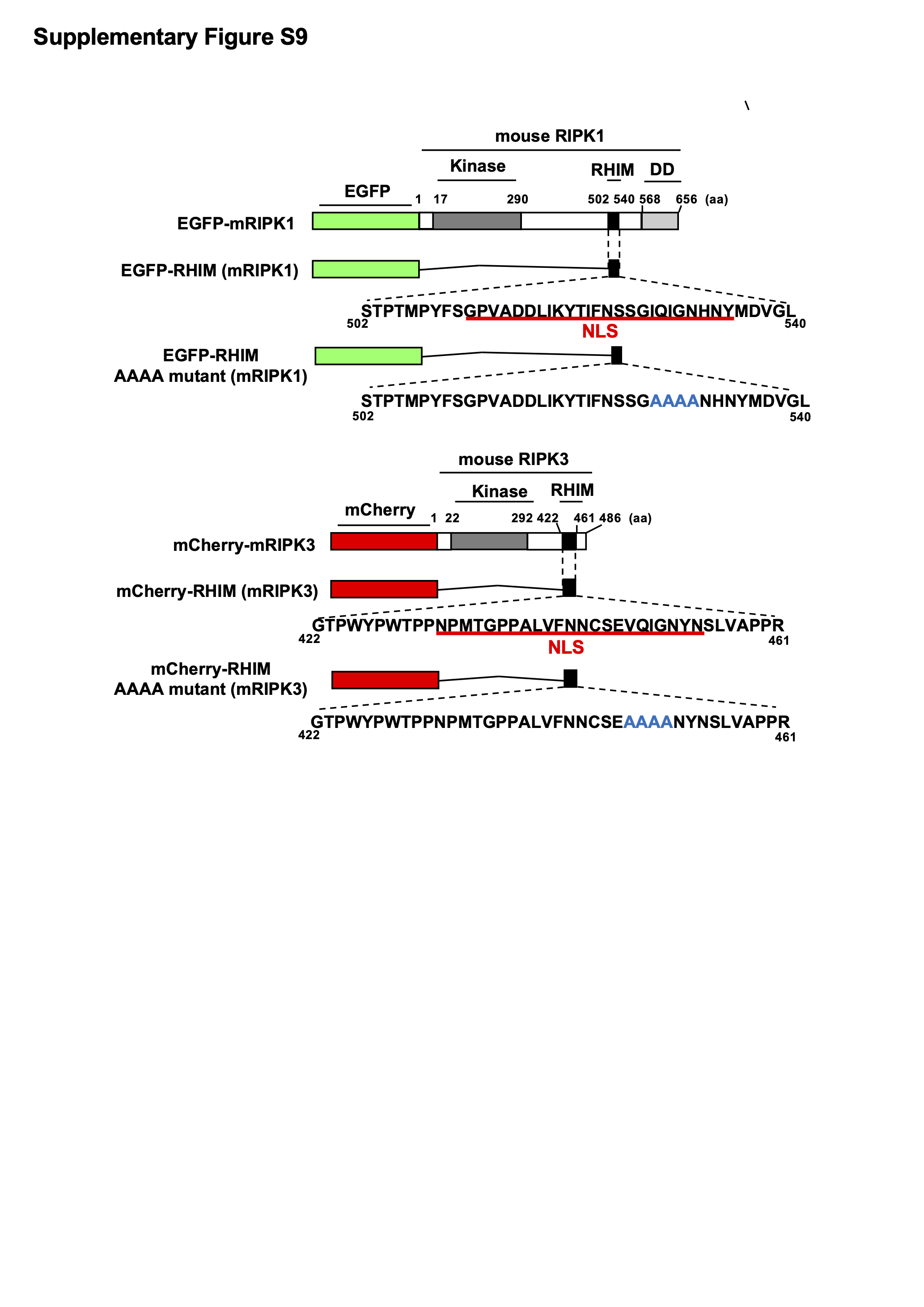

Supplement: Supplementary file 11 — Supplementary Fgiure S9 [file 41418_2019_434_MOESM11_ESM.tif]

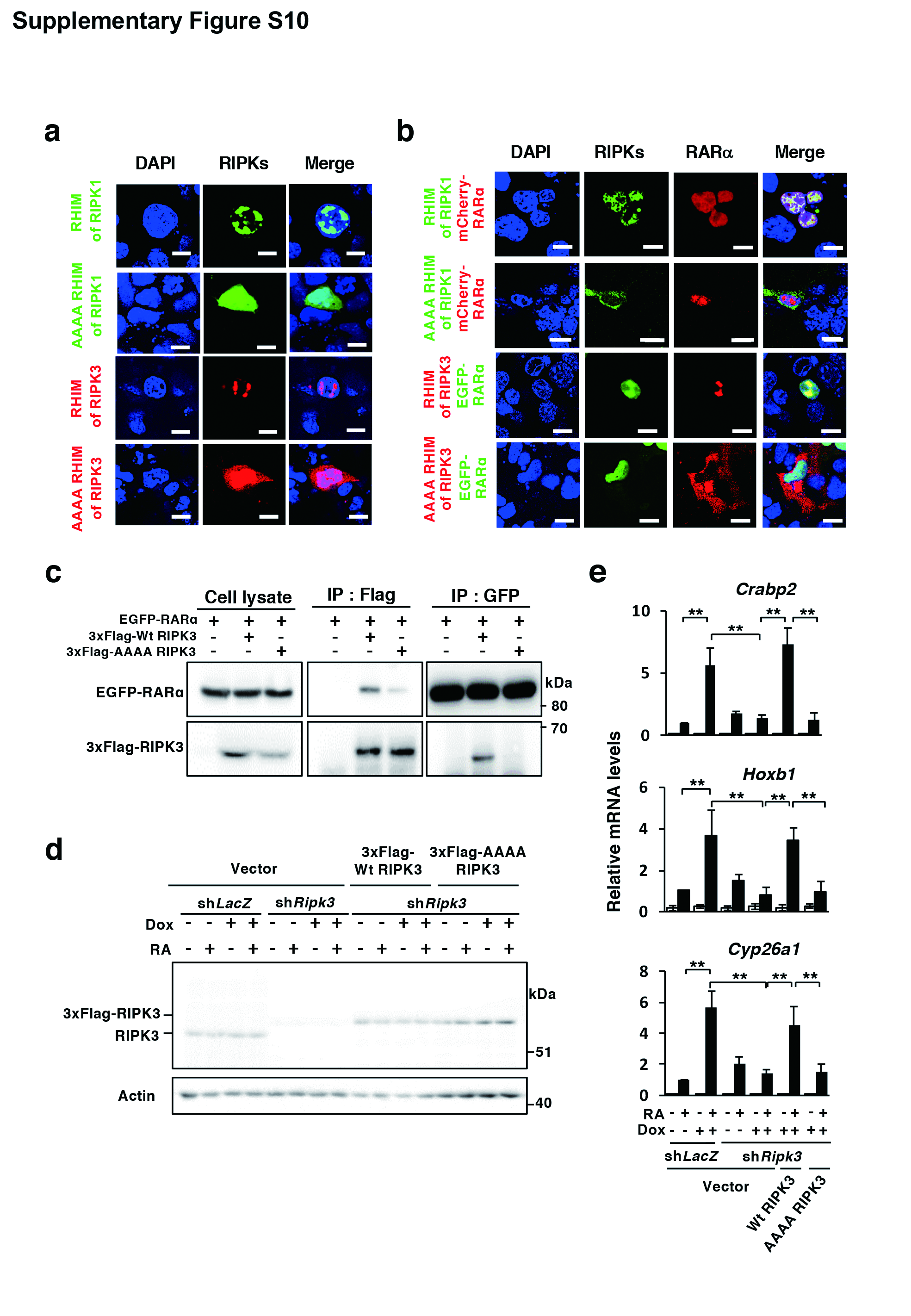

Supplement: Supplementary file 12 — Supplementary Fgiure S10 [file 41418_2019_434_MOESM12_ESM.tif]

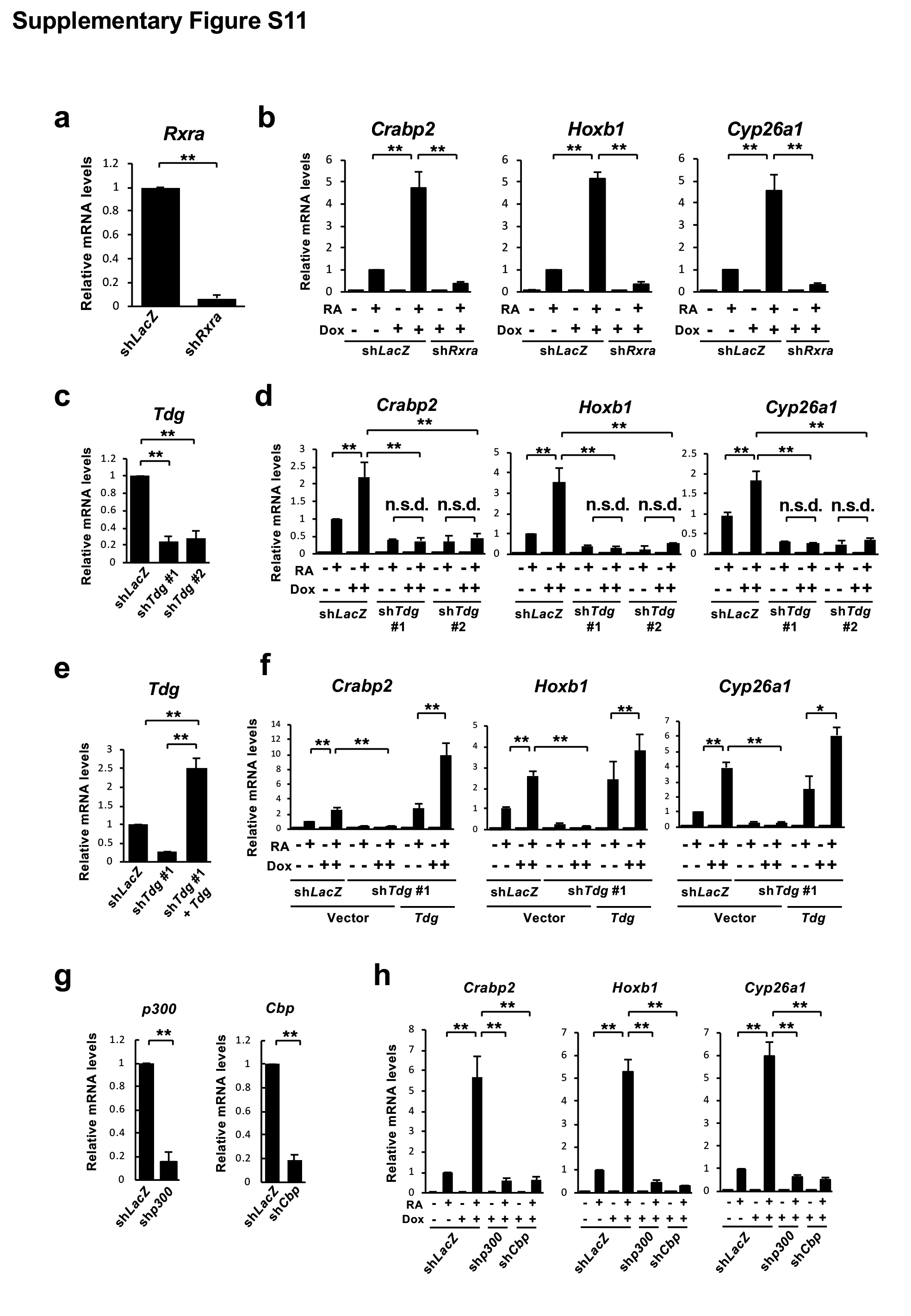

Supplement: Supplementary file 13 — Supplementary Fgiure S11 [file 41418_2019_434_MOESM13_ESM.tif]

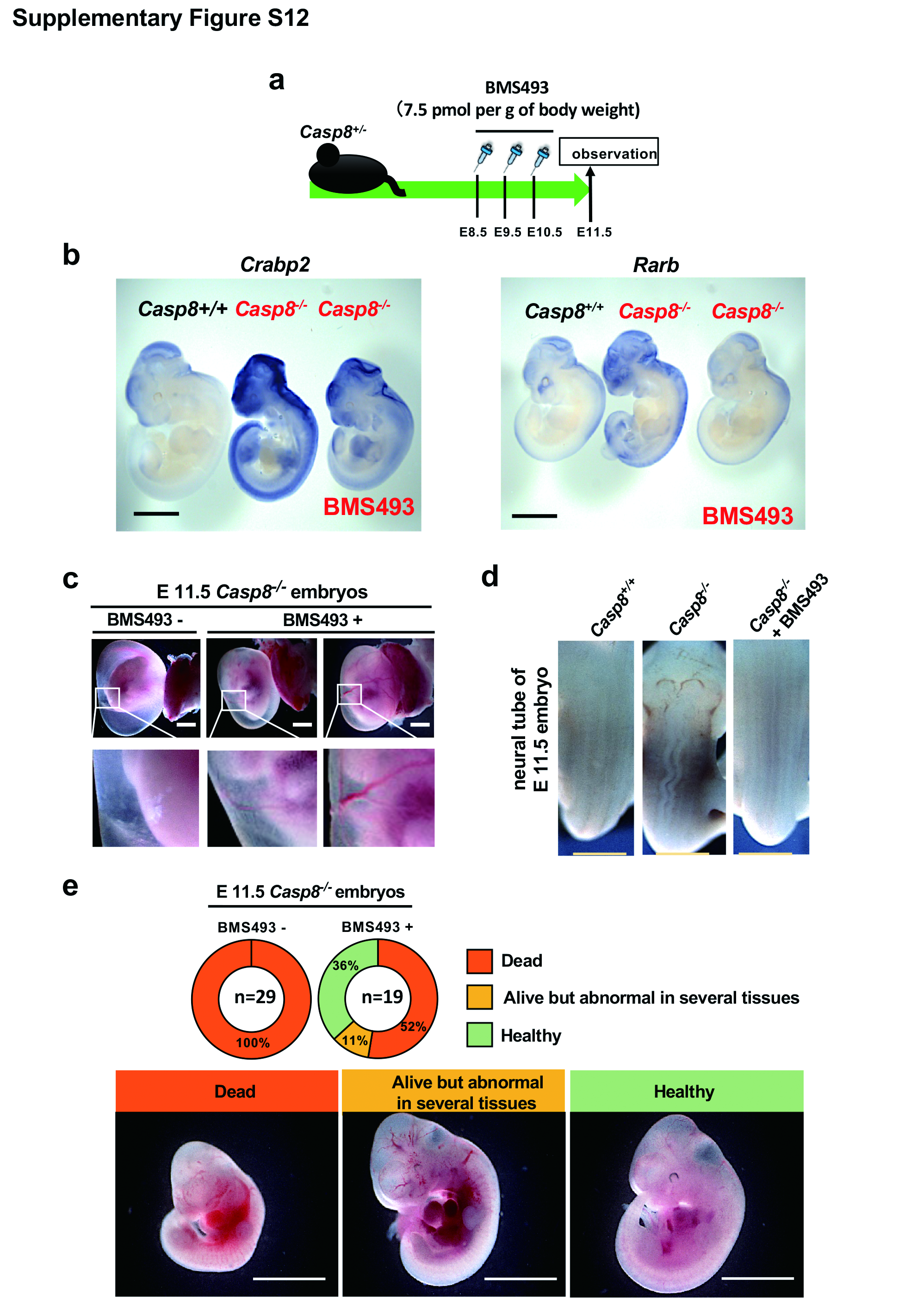

Supplement: Supplementary file 14 — Supplementary Fgiure S12 [file 41418_2019_434_MOESM14_ESM.tif]

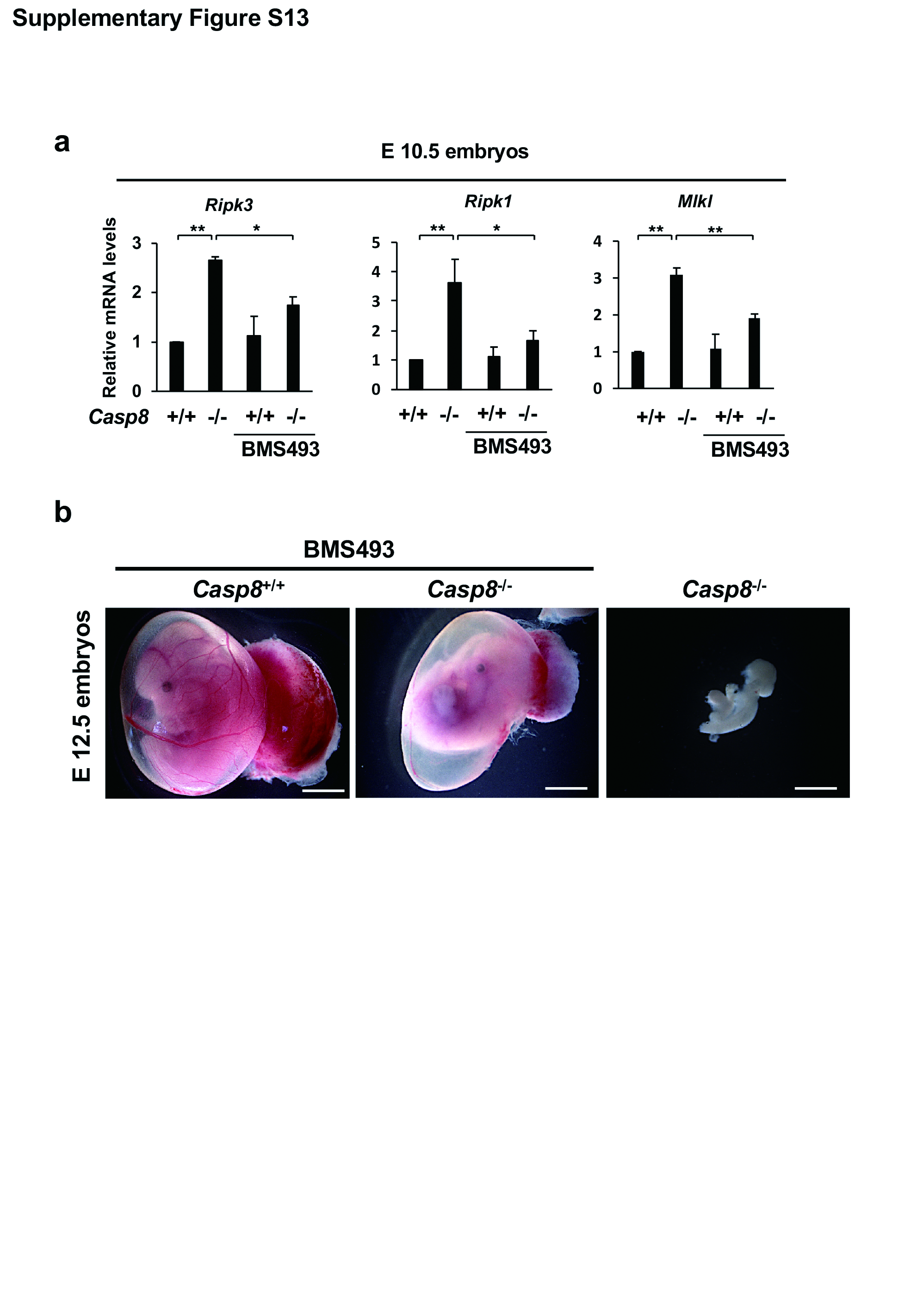

Supplement: Supplementary file 15 — Supplementary Fgiure S13 [file 41418_2019_434_MOESM15_ESM.tif]

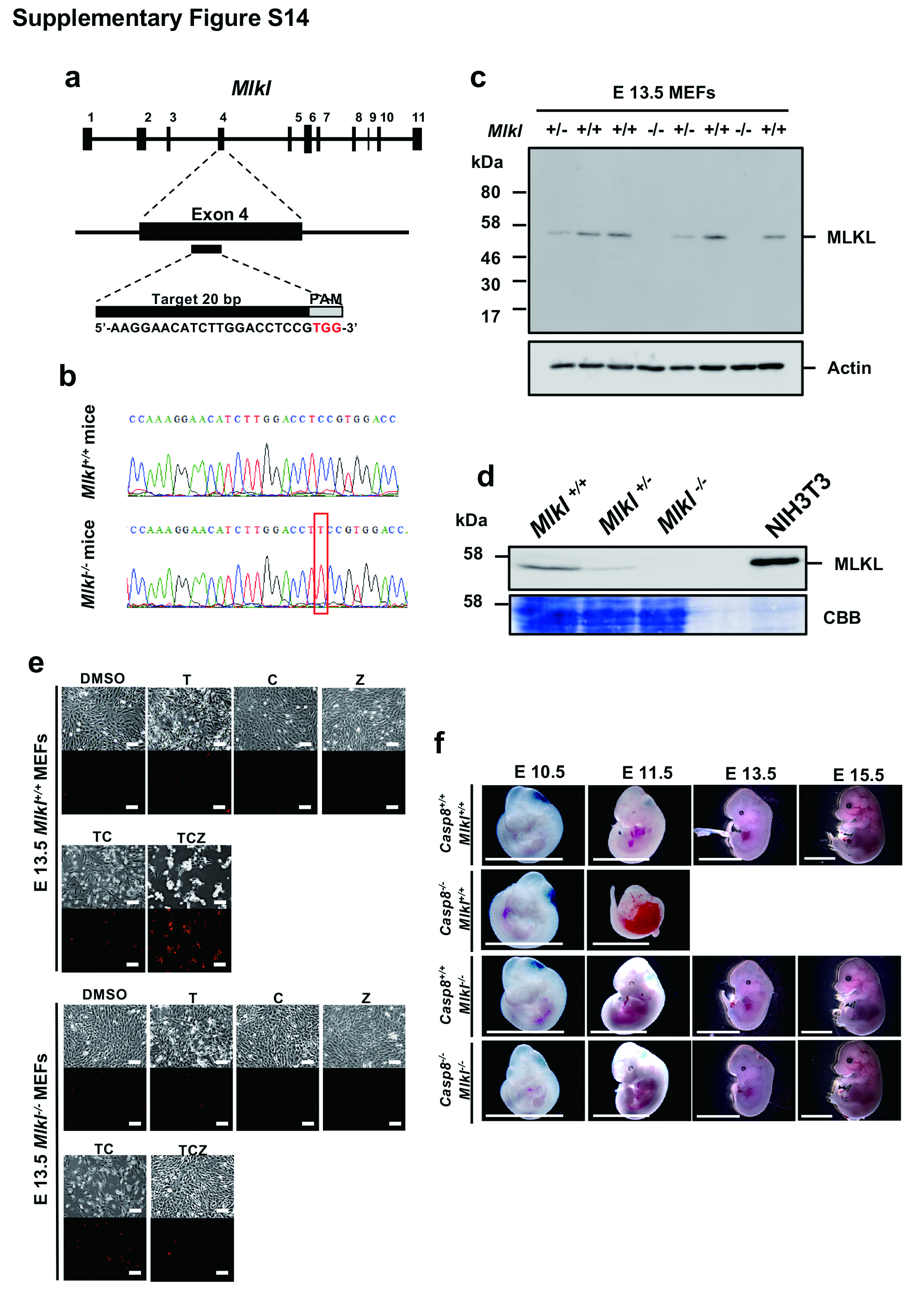

Supplement: Supplementary file 16 — Supplementary Fgiure S14 [file 41418_2019_434_MOESM16_ESM.tif]

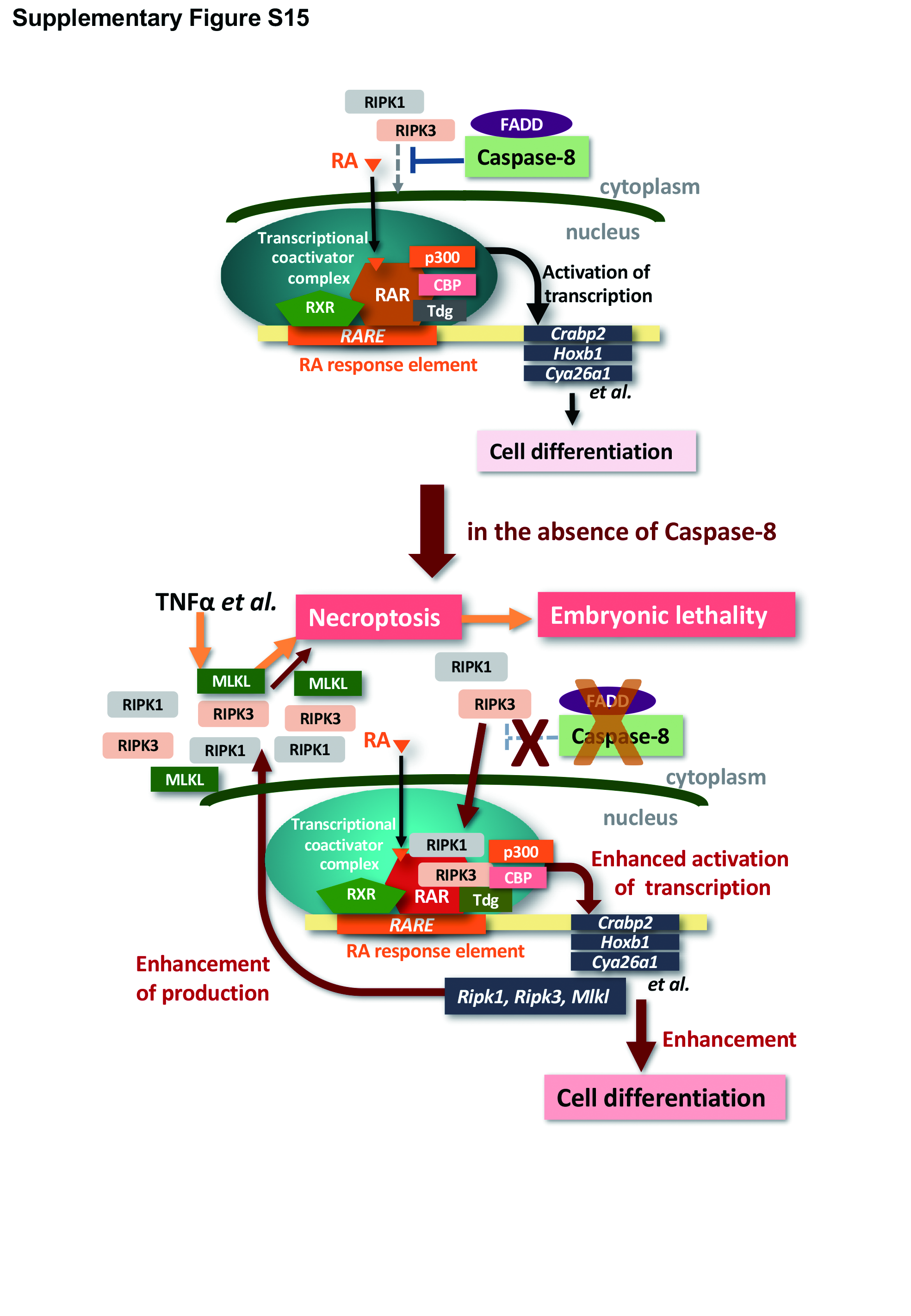

Supplement: Supplementary file 17 — Supplementary Fgiure S15 [file 41418_2019_434_MOESM17_ESM.tif]
